# A full-body transcriptome and proteome resource for the European common carp

**DOI:** 10.1186/s12864-016-3038-y

**Published:** 2016-09-02

**Authors:** I. C. R. M. Kolder, S. J. van der Plas-Duivesteijn, G. Tan, G. F. Wiegertjes, M. Forlenza, A. T. Guler, D. Y. Travin, M. Nakao, T. Moritomo, I. Irnazarow, J. T. den Dunnen, S. Y. Anvar, H. J. Jansen, R. P. Dirks, M. Palmblad, B. Lenhard, C. V. Henkel, H. P. Spaink

**Affiliations:** 1 Institute of Biology Leiden, Leiden University, Sylvius Laboratory, Sylviusweg 72, 2300 RA Leiden, The Netherlands; 2 Leiden Institute of Advanced Computer Science, Leiden University, Niels Bohrweg 1, 2333 CA Leiden, The Netherlands; 3 Center for Proteomics and Metabolomics, Leiden University Medical Center, 2300 RC Leiden, The Netherlands; 4 Computational Regulatory Genomics, MRC Clinical Sciences Centre, Faculty of Medicine, Imperial College London, Hammersmith Hospital Campus, Du Cane Road, London, W12 0NN UK; 5 Cell Biology and Immunology group, Department of Animal Sciences, Wageningen University, P.O. Box 338, 6700 AH Wageningen, The Netherlands; 6 Faculty of Bioengineering and Bioinformatics, Lomonosov Moscow State University, 119991, GSP-1 Moscow, Russia; 7 Laboratory of Marine Biochemistry, Department of Bioscience and Biotechnology, Kyushu University, Fukuoka, 812-8581 Japan; 8 Laboratory of Comparative Immunology, Department of Veterinary Medicine, Nihon University, Kameino 1866, Fujisawa, Kanagawa 252-0880 Japan; 9 Polish Academy of Sciences, Ichthyobiology and Aquaculture Unit, Gołysz Zaborze, Kalinowa 2, 43-520 Chybie, Poland; 10 Leiden Genome Technology Center, Human and Clinical Genetics, Leiden University Medical Center, Leiden, The Netherlands; 11 ZF-screens B.V., J.H, Oortweg 19, 2333 CH Leiden, The Netherlands

**Keywords:** *Cyprinus carpio*, Genome, Transcriptomics tissue-specific expression, RNA-Seq, Proteomics

## Abstract

**Background:**

The common carp (*Cyprinus carpio)* is the oldest, most domesticated and one of the most cultured fish species for food consumption. Besides its economic importance, the common carp is also highly suitable for comparative physiological and disease studies in combination with the animal model zebrafish (*Danio rerio*). They are genetically closely related but offer complementary benefits for fundamental research, with the large body mass of common carp presenting possibilities for obtaining sufficient cell material for advanced transcriptome and proteome studies.

**Results:**

Here we have used 19 different tissues from an F1 hybrid strain of the common carp to perform transcriptome analyses using RNA-Seq. For a subset of the tissues we also have performed deep proteomic studies. As a reference, we updated the European common carp genome assembly using low coverage Pacific Biosciences sequencing to permit high-quality gene annotation. These annotated gene lists were linked to zebrafish homologs, enabling direct comparisons with published datasets. Using clustering, we have identified sets of genes that are potential selective markers for various types of tissues. In addition, we provide a script for a schematic anatomical viewer for visualizing organ-specific expression data.

**Conclusions:**

The identified transcriptome and proteome data for carp tissues represent a useful resource for further translational studies of tissue-specific markers for this economically important fish species that can lead to new markers for organ development. The similarity to zebrafish expression patterns confirms the value of common carp as a resource for studying tissue-specific expression in cyprinid fish. The availability of the annotated gene set of common carp will enable further research with both applied and fundamental purposes.

**Electronic supplementary material:**

The online version of this article (doi:10.1186/s12864-016-3038-y) contains supplementary material, which is available to authorized users.

## Background

The common carp (*Cyprinus carpio)* is the oldest, most domesticated and one of the most cultured fish species for food consumption, angling purposes and expensive species as ornamental fish. Geographical and reproductive isolation resulted in the formation of two subspecies; European common carp (*Cyprinus carpio carpio*) in the West and East-Asian common carp (*Cyprinus carpio hematopterus*) in the East [1]. It is closely related to zebrafish (*Danio rerio*), a commonly used animal model to study human disease [2–4], with both lineages diverging approximately 11–21 million years ago [5, 6]. Because of its large body size, a single animal can yield sufficient amounts of tissue from its organs and blood for extensive genomic, proteomic and metabolic studies without compromising to possible contaminations with neighboring tissues. Especially for small organs, the carp also offers far better possibilities than zebrafish for obtaining tissues that are well-separated from other tissues. Furthermore, carp itself, besides being an important edible species and offering complementary benefits to the closely-related zebrafish, is emerging as a highly useful animal model on its own, providing valuable information for questions on physiology, genetics, immunology and disease [1]. For instance, common immune genes that were notably lacking in zebrafish were shown to be present in the carp genome [7].

Recently, several full genome assemblies of carp species have become available [8, 9]. These studies confirm the duplicate nature of the carp genome with respect to zebrafish and several other teleosts, with the carp lineage having experienced a recent (8 Mya) allotetraploidization event [10, 11]. The carp genome consists of 50 chromosome pairs, in contrast to 25 for zebrafish [9]. Between carp and zebrafish, extensive conservation of synteny remains [8, 9]. Curiously, the carp genome is of approximately equal size to the zebrafish genome, the result of less intra- and intergenic repetitive content [8]. Further comparative genomics and transcriptomics studies using Hungarian, North American and Chinese carp strains have provided insights into the species structure, and identified genes associated with skin color and scale phenotypes [9]. However, further analyses of carp transcriptomes are not yet abundant, with an emphasis on embryonic samples and mixed tissues [8, 9].

A major drawback of the genomic information on carp is the lack of a genome-based annotation of all gene predictions in public databases. As a result, the accessibility of carp data has been limited to specialist analyses. Here, we describe the generation of an annotated gene set of *C. carpio* based on published and new genomic data, generated by the sequencing of long DNA fragments using Pacific Biosciences technology. We use this annotated set to investigate tissue-specific gene expression, based on RNA-Seq data generated for dozens of distinct tissue samples. In addition, we have performed deep proteomic analyses of several tissues for comparison with the transcriptome data.

In order to assess the common carp as more than only an important edible species, we aimed to validate carp as an animal model complementary to the zebrafish used for human disease studies, and investigate the relationship between these two closely-related cyprinid species by comparing the expression of tissue-specific genes in carp and predicted tissue-specific expression in zebrafish. Together, access to a well-annotated genome and the whole animal approach to tissue gene expression provide new resources which will substantiate research on common carp.

## Methods

### Carp genome

#### Carp fish line

R3 x R8 are the hybrid offspring of a cross between carp of Hungarian origin (R8 strain) and of Polish origin (R3 strain) [12], each of which are kept purebred by single brother-sister matings [13–15]. A cross between single R3 and R8 individuals purebred for five generations led to a base population for artificial reproduction by induced gynogenesis and subsequent production of a homozygous all-female clonal line, which was sampled for genomic DNA to construct genomic libraries [12]. A cross between single R3 and R8 individuals purebred for 11 generations led to a base population for sampling of organs for RNA sequencing. Genomic DNA was used for construction of genomic libraries as described [8]. The breeding of adult fish was approved by the local animal welfare committee (DEC) of Wageningen University. All protocols adhered to the international guidelines specified by the EU Animal Protection Directive 2010/63/EU.

#### CLIP-PE sequencing

CLIP-PE Libraries were made mostly according to the protocol published by Peng et al. [16]. DNA was sheared using a Covaris g-TUBE by centrifuging for 2 × 1 min at 8000 rpm in an Eppendorf 5414R centrifuge. The DNA was subsequently run on a 0.6 % agarose gel in TAE buffer to isolate the desired range of fragments. The Qiagen QIAexII kit was used to isolate the DNA from the gel. To repair the DNA damage a PreCR incubation was done according to the manufacturers description (NEB, Ipswich MA, USA). After Ampure XP purification the DNA fragments were end repaired and A-tailed using the NEBNext DNA library prep reagent set (NEB). Adapters containing LoxP sites were ligated to the DNA using the Quick ligase kit (NEB). Adapter ligated fragments were then circularized using Cre recombinase (Life technologies). After circularization the linear DNA fragments were digested using Plasmid safe ATP-dependent DNase (Epicentre). Circularized DNA fragments were digested using *Nla*III (NEB). After digestion the reaction volume was increased to promote self-ligation and DNA fragments were again circularized using T4 ligase. Linear DNA fragments were removed using Plasmid safe ATP-dependent DNase. A PCR with Illumina PE PCR primers was performed to amplify the fragments and to add Illumina flow cell compatible ends to the DNA fragments. The libraries were paired end sequenced using Illumina Hiseq 2500 technology with a read length of 51 nt.

#### Long read sequencing

Genomic DNA for Pacific Biosciences sequencing was isolated from nucleated red blood cells, from a single adult female carp from a homozygous clonal common carp line (R3R8 69–45) described by Henkel et al. [8]. DNA was isolated using the Qiagen Blood and Tissue DNeasy kit according to manufacturer’s manual (Qiagen, Hilden, Germany).

The isolated DNA was fragmented with gTUBEs (Covaris) and end-repaired. SMRTbell DNA template libraries (insert size of 20Kb) were prepared according to the manufacturer’s instruction. Sequencing of ten SMRT cells was performed on the Pacific Biosciences RSII platform using the Magbead loading protocol and P5-C3 chemistry. Sequencing reads had an N50 of 11.9 kbp. The total number of bases produced was 6.8 Gbp, which is the equivalent of 4.5× coverage of the genome.

#### Assembly

The genome was assembled by integrating the contigs generated previously [8] with the PacBio sequencing reads and the three CLIP-PE libraries with insert sizes of 5, 6.5, and 7.5 kbp. The paired-end data was used to construct scaffolds using SSPACE [17]. Subsequently, PBJelly [18] was used to scaffold fragmented contigs and fill the gaps using long and single-molecule PacBio reads. Gaps that were covered by at least 5 PacBio reads were filled by the consensus sequence. Otherwise, Ns were used to fill gaps between contigs that were linked by less than 5 PacBio reads.

#### Annotation

As the first step of genome annotation, we applied repeat analysis with RepeatMasker (v4.0.5) [19] and the RMBlast engine [19]. In addition to Repbase (v20140131) [20], we constructed a *de novo* transposable element library for the carp genome from RepeatModeler (v1.0.8). The genome was then annotated using the MAKER pipeline (v2.31.8) [21]. The construction of gene models was based on three sources of information: *ab initio* gene prediction, homologs-based evidence and expression evidence from a *de novo* transcriptome assembly. The *ab initio* gene prediction software AUGUSTUS (v3.1.0) [22], with trained gene model parameters, was used to predict genes from the repeat-masked genome. For homologs-based prediction, the zebrafish protein sequences from Ensembl version 77 were collected to build a protein database for BLASTX (v2.2.27) to align genome sequences to the corresponding protein sequences. To annotate the putative gene functions, we conducted a BLASTP (v2.2.27) search for the identified protein sequences against the Swiss-Prot database with the E-value of 1e-5. The protein domain information and GO category classification were produced from InterProScan (v5.11-51.0) [23].

### RNA-sequencing

#### Sample collection

**Fig. 1:**
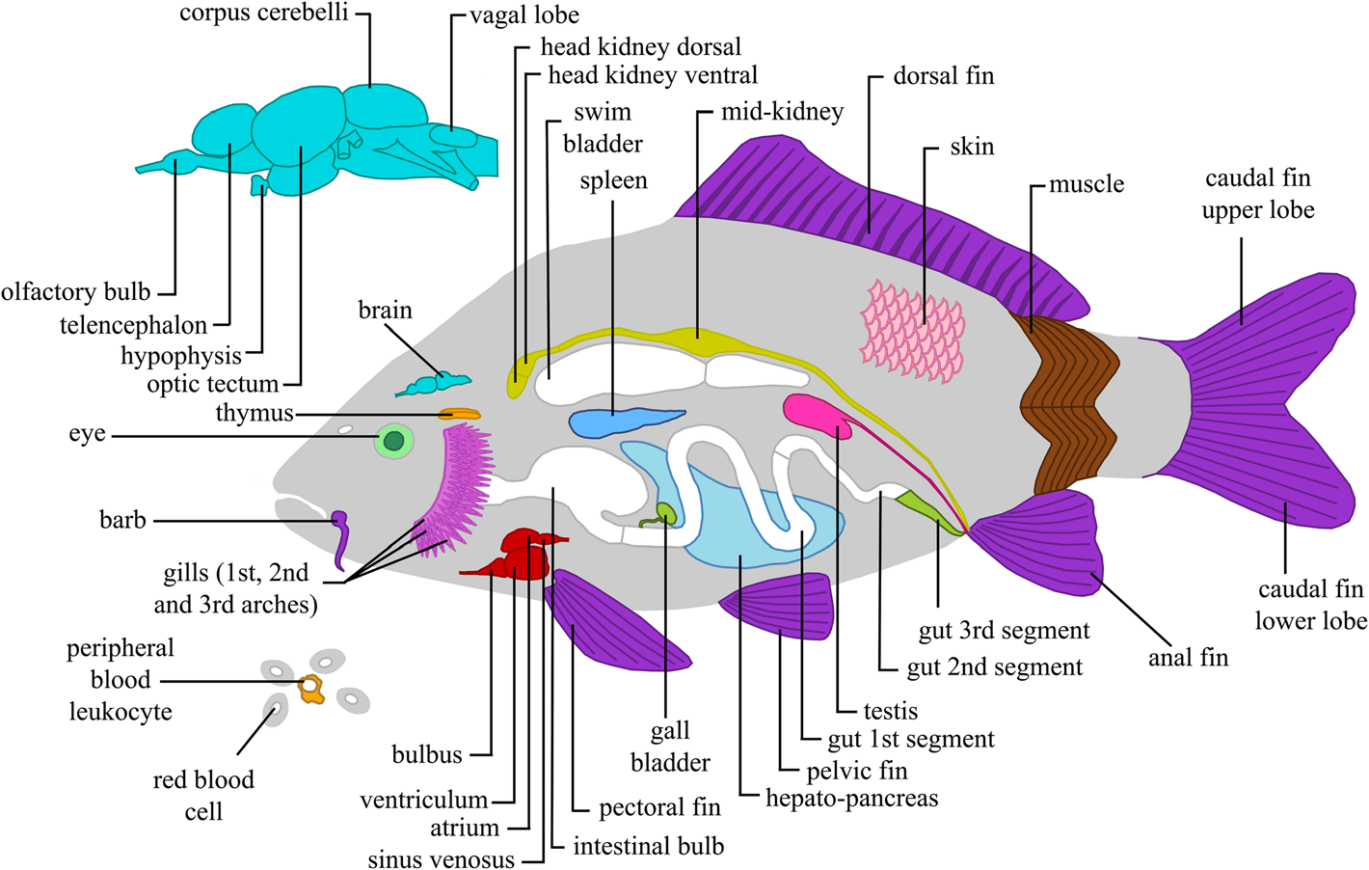
A cross-section of the carp organs sampled for this study. The different tissues were colored according to the 16 groups, to match the coloring of the following figures. A full list is available in Additional file 1: Table S1

From common carp with a comparable genetic background (after 11 generations of brother-sister matings) as the carp used for the carp genome [8], we used four healthy adult F1 hybrids. Carp specimens (1 male, 3 females) which were dissected into 89 samples from 19 different tissues (Additional file 1: Table S1). Additionally, two pools of embryos at 5 days post fertilization (dpf) were used. An overview of tissues collected is depicted in Fig. 1. Tissue samples were stored in RNAlater (Life Technologies, Carlsbad, USA), after which RNA was isolated using the Qiagen Blood and Tissue DNeasy kit according to manufacturer’s manual (Qiagen, Hilden, Germany).

#### Illumina sequencing and processing

A library was made with the TruSeq Stranded total RNA library prep kit according to the manufacturer’s description (Illumina inc, San Diego, USA). Both paired end and single libraries were sequenced using an Illumina HiSeq 2500 according to the manufacturer’s description. Illumina software (HCS) was used for basecalling. Tophat version 2.0.5 [24] was used to align the reads to the reference genome. For each read pair, secondary alignments (which meet alignment criteria but are less likely to be correct) were filtered out using samtools version 0.1.18 [25]. For each predicted gene, read counts were obtained from the alignment file using HTSeq-count version 0.5.3.p9 [26] using the ‘intersection-strict’ setting to ignore reads not aligning to annotated exons.

#### Data analysis

**Fig. 2:**
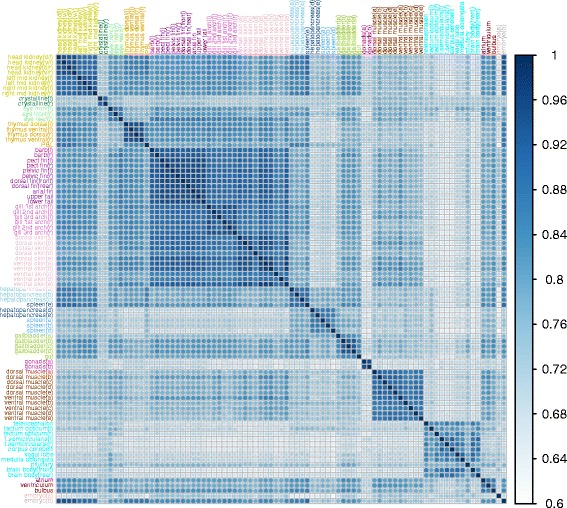
Correlation plot using the Spearman correlation test on the RPKM rank-normalized data (*n* = 87) after mixed/outlier samples were removed. The different tissues were colored according to the 16 groups

Data quality was assessed using the statistical package R [27]. Most importantly, raw RNA-Seq counts need to be normalized to correct for sequencing depth and (optionally) transcript length. Dividing counts by simple sequencing depth (the total number of reads) can lead to inaccurate results [28], as it is strongly influenced by the most abundantly expressed genes. For example, extreme expression of a single gene (e.g. hormone-encoding genes [29]) will strongly affect the expression measures of all other genes if naïve CPM (counts per million) or RPKM (reads per million per kilobase) normalization is used. Alternatively, empirical estimates of sequencing depth (available in the edgeR package, v. 3.12.0 [30]) are commonly used. However, like CPM and RPKM, these assume samples to be similar and perform poorly on our multi-tissue data (Additional file 2: Figure S1A-B, log10 scale). We therefore ranked the count data and normalized between samples by considering CPM- or RPKM-based expression based on the ranks instead of actual counts. Using this procedure, expression in all samples can be made comparable (Additional file 2: Figure S1C-D, log10 scale). A correlation matrix was calculated on the RPKM data with a Pearson correlation test (Additional file 3: Figure S2). After removal of outliers a correlation matrix was calculated on the rank-normalized data of the remaining samples with a Spearman correlation test (Fig. 2). The correlation matrices were plotted using the corrplot package (v0.73) [31]. Tissue samples were clustered according to the correlation plots (Additional file 4: Table S2). For each of these tissue groups the mean of the samples in that group were taken for each gene. On these means, gene clusters were calculated using k-means clustering using the MultiExperiment Viewer (MeV version 4.9) [32]. The genes in the resulting gene clusters were ranked on the mean of the overall expression. The top 10 expressed genes were plotted in a heatmap using gplots (v2.17) [33]. The software program Last [34] was used to align the Chinese Songpu strain to our assembly at DNA level. Post-processing of Last results produces the actual whole-genome alignments. In this first step, original alignment blocks were chained together if their genomic locations in both genomes are close enough. Then netting process chose the best match in each region for the reference species. The post-processing was done by UCSC Kent utilities. InParanoid [35] was used to generate the orthologs mapping between carp and zebrafish.

### Proteomics

#### Protein isolation

Proteins were extracted from heart, hepatopancreas and kidney tissue using 20 mM NaCl, 20 mM Tris-HCl pH 8.2, 50 U/mL Benzonase, 2 mM MgCl_2_, and protease inhibitors (Roche), followed by homogenization with zirconium oxide (0.5 mm) beads using a Bullet Blender (Next Advance) at speed 8 for 3 min. The samples were placed at 95 °C for 5 min, and centrifuged for 30 min at 4 °C and 16,0000 × g. The supernatant was transferred into a fresh tube and the pellet used to perform a second extraction by adding 1 % SDS, 50 U/mL Benzonase, 2 mM MgCl_2_, and protease inhibitors, followed by the same extraction procedure. Protein concentration of both extracts were determined using a BCA assay (#23235, Bio-Rad). Approximately 40 μg of both extracts was loaded on Novex 4–12 % Bis-Tris gels (NuPAGE, Invitrogen) after adding 5 μL NuPAGE LSD Sample buffer (Invitrogen), and ran at 200 volt using MOPS SDS running buffer (Invitrogen) and an XCell Sure Lock Mini-Cell (Invitrogen) to fractionate proteins from each tissue solution. The gels were stained overnight in colloidal blue staining (LC6025 kit, Invitrogen) solution (55 mL deionized water, 20 mL methanol, 20 mL Invitrogen stain A, and 5 mL Invitrogen stain B) on a shaker at room temperature. Gels were washed twice in deionized water and scanned on an OptiGo UV imager (Isogen Life Science). The gel lanes were cut in 48 identical pieces (Lane Picker, The Gel Company) and transferred to a 96-well plate using tweezers. Preparation for in gel digestion was performed at room temperature unless mentioned differently, and all washed with 100 μL solution [36].

#### LC-MS/MS

Peptide separation and MS/MS measurement were performed as described previously [37]. In summary, to separate all peptides from each individual gel lane, ten μL of each sample was loaded and desalted on C18 PepMap 300 Å precolumn (Thermo Scientific) and separated by reversed-phase liquid chromatography using two identical 150 mm 0.3 mm–i.d. ChromXP C18CL, 120 Å columns (Eksigent) which were coupled parallel and connected to a split less NanoLC-Ultra 2D plus system (Eksigent) with a linear 90-minute gradient from 4 to 35 % acetonitrile in 0.05 % formic acid and a constant (4 μL/minute) flow rate. The separated peptides were then inserted into an amaZon speed ETD ion trap (Bruker Daltonics) with an Apollo II ESI source to which the LC was coupled. Up to 10 abundant multiply charged species in *m*/*z* 300–1300 were selected for collisionally induced dissociation MS/MS after each MS scan, and excluded for 1 min after having been selected twice. The systems were controlled by HyStar 3.2 and trapControl 7.1, for the LC and ion trap respectively.

#### Data analysis

CompassXport 3.0.5 was used to convert raw ion trap data into mzXML format [38]. Spectra were matched to peptides using the program X!Tandem [39] of the Trans Proteomic Pipeline, searching each spectrum against our carp database. These peptide spectrum matches (PSMs) were validated by Peptide Prophet [40, 41] resulting in protein identifications exported with a minimum probability of 0.95 and a global false-discovery rate (FDR) of 1 %. Proteomics data was plotted against the RNA-Seq data on a log10 scale in R (ggplot2) [42]. Correlations were calculated with the Spearman correlation method in R.

### Organ data viewer

The top 10 most highly expressed genes from cluster 6, 11, 12 and 15 were selected. The expression levels of these genes in different organs and tissues were visualized using a script written in R [27], using the maps [43], maptools [44], sp, lattice, laticeExtra [45] and colorspace [46] packages as in a previous presentation of geographic data [47]. We created cartoon-like carp shapefiles in QGIS [48] to visualize the carp organs in a spatial context. Each organ or tissue was defined as a polygon and linked with the expression data. Tissues that had many different randomly dissected parts (skin and muscle), were represented with the mean value of all the different sections. For each gene, RNA-Seq expression levels were linked to the indices locating the respective organ or tissue. Finally, this table was used to generate a heat map in a red color scale to represent the levels of expression on a logarithmic scale.

## Results and discussion

### Carp genome assembly

In order to enable isogenic mapping of RNA-Seq data, we have improved the genomic sequence of carp [8] using long-range sequencing information. Using the same DNA sample employed for the initial assembly [8], we have prepared three paired-end libraries using the Cre-LoxP Inverse PCR (CLIP) approach [16] with target insert sizes of 5, 6.5, and 7.5 kbp. For each library 4.5 million read pairs were obtained. In addition, we have sequenced 6.8 Gbp of DNA isolated from nucleated red blood cells using Pacific Biosciences long read technology. All sequencing data was included in an updated *de novo* genome assembly. The new assembly is 1.38 Gbp in length, and has a scaffold N50 of 66.7 kbp. This is considerably less than the 1.69 Gbp genome reported for mirror carp strain Songpu [9]. In Table 1 we have compared several quantitative features of our genome assembly and that of the Songpu strain. The software program Last [34] was used to align the Chinese Songpu strain to our assembly at DNA level. The results show that the assemblies preserve syntenic relationships for 1.13Gb, which constitutes 82 % and 67 % of our assembly and the published assembly of Songpu, respectively.

**Table 1:** Comparison of features of the common carp genomic assembly used in this paper and that of common carp strain Songpu [9]

Data set	Genome size	Scaffolds	N50	GC content	Predicted genes	Median gene span	Predicted exons (total/per gene)	Repeats
This work	1.38Gb	80273	67 kb	37 %	50527	8316 bp	387245/7.7	32 %
Xu et al., 2014 [9]	1.69Gb	2503	1 Mb	36.30 %	52610	11980 bp	390620/7.5	31 %

At present, it is not possible to determine the exact cause of the discrepancy in assembly length. One possible explanation is that our genome is based on homozygous double-haploid carp, whereas the Songpu genome is based on a heterozygous individual. Variations can be expected since high phenotypic and high genomic variation are compatible with ongoing re-diploidization following the carp-specific genome duplication. The recently published genome of the Atlantic Salmon suggests re-diploidization following genome duplication is accompanied by substantial genomic instability [49]. As the carp-specific duplication is much more recent than the salmonid-specific one (~80 Mya), carp genomes might still be experiencing high levels of genomic rearrangements.

However, the discrepancy between the genome assemblies of common carp strains should be attributed at least in part to the sequencing technologies employed. Both assemblies are primarily based on short-read assemblies, which are not optimally suitable for obtaining precise long-range genomic distance information. Here, we have already demonstrated the usefulness of low-coverage Pacific Biosciences sequencing for augmenting genome assemblies. It will therefore be interesting to further enhance and verify both genome assemblies using emerging single-molecule technologies, such as Oxford Nanopore long read sequencing, or BioNano Genomics optical mapping [50].

### Tissue-specific carp transcriptomes

We have sequenced cDNA obtained from 89 tissues of carp and two embryos, with the aim of generating a comprehensive atlas of the carp transcriptome. These data were used for supporting gene prediction and quantification of gene expression.

Genes were predicted both *de novo* and using RNA-Seq data, as described in the material and methods section. We could predict the presence of 50527 genes, which is similar to the 52610 genes predicted for strain Songpu [9]. The predicted genes are all made available in the NCBI database, thereby enormously expanding the previous set of a few thousand annotated genes of common carp.

Subsequently, we quantified the expression of all genes in all 91 samples. For initial data exploration, we used these counts to generate a correlation matrix showing the similarity of transcriptomes between all samples across all genes (Additional file 3: Figure S2). This analysis revealed four outlier samples (left eye rear, optical nerve, gallbladder (e) and swim bladder rear), for which the global expression pattern did not match that of replicates but instead appeared intermediate between tissues. In all cases, this could be explained by slight contamination with neighboring tissues during dissection. This is a known issue when dissecting organ tissue in fish [51, 52].

As expected, the initial analysis also revealed that specialized tissues vary widely in gene expression patterns. This poses a problem, as nearly all RNA-Seq analyses implicitly or explicitly assume that samples are comparable (e.g. treated and non-treated). Therefore, for clustering purposes, we normalized between samples using expression ranks rather than values (see Materials and Methods). This approach, routinely used in microarray analyses, can be employed to make intrinsically dissimilar samples comparable [53]. However, when comparing across samples, the resulting expression can only be interpreted qualitatively rather than quantitatively (e.g. ‘high in liver/low in eye’ instead of ‘higher in liver than in eye’). For display purposes, we therefore used RPKM-expression values, which allow for within-sample comparison of expression levels.

Subsequently, we clustered the ranked data for the remaining 87 samples in a correlation matrix as before (Fig. 2), confirming that the remaining tissues cluster with their own tissue type. To investigate tissue - specific RNA expression, all the samples were grouped by tissue based on the correlation between the samples. We found two samples of hepatopancreas (d,e) that clustered more like spleen tissue and one spleen(e) sample that clustered more like hepatopancreas tissue. Due to their close proximity we assume this is due to the dissection of the tissues. We grouped these three samples as a mixed hepatopancreas/spleen group.

**Fig. 3:**
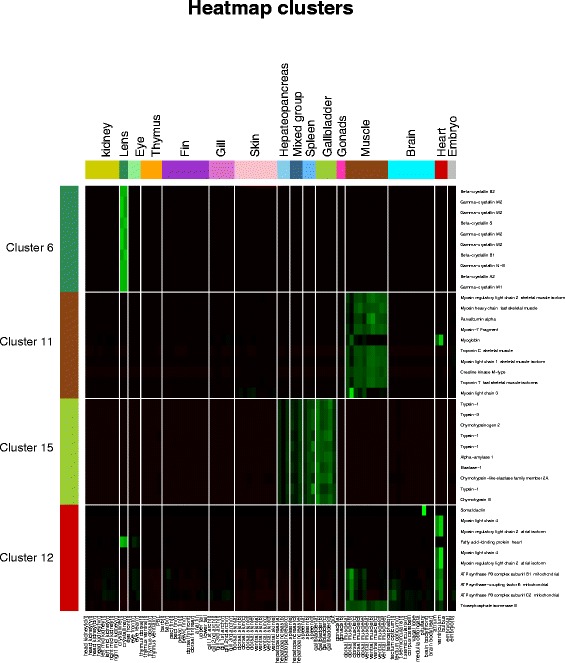
Heatmap on the RPKM-normalized data. Four of the gene clusters (cluster 6-lens, 11-muscle, 12-heart and 15-gallbladder) are visualized, depicting the top 10 highest expressed genes in that respective cluster. The different tissues were colored according to the 16 groups defined in Fig. 1 (The other gene expression clusters are depicted in Additional file 5: Figure S3)

Based on the correlation plot (Fig. 2), the tissues were categorized into 16 groups. For each gene the mean of all the samples in one group was taken. In these 16 tissue groups, we investigated possible gene clusters specific for one type of tissue. Using the k-means clustering function in Mev [32, 54], all genes were divided into 16 clusters on ranked data. Within these 16 gene clusters the genes were ranked based on the sum of the overall expression in the samples. We used the top 10 genes to create a heatmap (Additional file 5: Figure S3). We highlight four of these clusters (cluster 6, 11, 12 and 15) in Fig. 3. Gene cluster 6 exhibits highly specific expression in lens tissue only. Gene clusters 11, 12 and 15 show a tissue-specific expression in muscle, heart and gallbladder, respectively. For each cluster, the most specific genes are functionally closely related. For example, the most abundant transcripts in cluster 6 exclusively encode crystallins, which are water soluble structural proteins present in the eye lens and cornea [55]. The beta and gamma crystallins are known to have very high homology between carp and zebrafish [56] and differ in their expression levels over the life span [57]. Similarly, the muscle-specific cluster (11) predominantly contains the myosin motor proteins essential for muscle contraction. Interestingly, several of these proteins seem to be skeletal muscle (myhz1.1) or heart specific (myl7). The latter is widely used as a heart tissue specific marker in zebrafish. Chymotrypsinogen 2, specific for the gall bladder and spleen (cluster 15) is known to be excreted in the gastrointestinal track in zebrafish, and in situ hybridization analysis of its homologue *ctrb1* shows it to be expressed in both the pancreas and gastrointestinal track in zebrafish [58].

Considering the close genetic relationship between the carp and zebrafish, we tested if the same genes expressed in different zebrafish tissues were also expressed in the carp. From ZFIN we collected lists of genes expressed in different zebrafish tissues, and removed the genes that were not found in our data. In zebrafish, the expression measured for liver and pancreas was combined to compare to our hepatopancreas sample of the carp. For skin we combined the genes found in zebrafish fin and tail tissue. For each tissue, the genes from ZFIN were compared to the genes expressed in the carp.

For the majority of tissues we find that 89.6–98.9 % of the genes expressed in zebrafish are also expressed in the same tissue in carp (Table 2). The hepatopancreas and spleen tissues have a lower match 85.2 % and 73.6 % respectively. As these tissues are known to be very variable between samples this lower match is not surprising and by removing the three potential mixed samples we have still managed to get a very high match with zebrafish expression.

**Table 2:** Gene expression comparison between carp and zebrafish tissues

Organ	Carp only	ZFIN only	Carp & ZFIN (% of ZFIN)
Kidney	17458	57 (7,1 %)	741 (92,9 %)
lens	14555	38 (6,2 %)	578 (93,8 %)
Eye	15041	116 (3,5 %)	3181 (96,5 %)
Thymus	18673	3 (3,8 %)	75 (96,2 %)
Fin	16868	39 (1,8 %)	2072 (98,2 %)
Gill	16964	80 (5,5 %)	1371 (94,5 %)
Skin	17931	26 (2,7 %)	947 (97,3 %)
Hepatopancreas	13826	253 (14,8 %)	1459 (85,2 %)
Spleen	14180	87 (26,4 %)	243 (73,6 %)
Gallbladder	18011	1 (3,0 %)	32 (97,0 %)
Gonads	17975	51 (6,1 %)	783 (93,9 %)
Muscle	16427	93 (5,7 %)	1535 (94,3 %)
Brain	15219	68 (1,7 %)	3871 (98,3 %)
Heart	15433	169 (10,4 %)	1457 (89,6 %)
Embryo	18431	11 (1,1 %)	1031 (98,9 %)

We compared the list of genes found to be expressed (per tissue) from the ZFIN database to the list of genes found to be expressed in the carp tissue of this study. The numbers given refer to the gene count found exclusively in carp (carp only), ZFIN (ZFIN only) or present in both carp and ZFIN. The overlap in percentage of the total number of ZFIN genes linked to these tissue is also shown

As the carp genome has experienced a recent duplication event since the split with zebrafish, a majority of genes have a duplicate in common carp [8]. It is of interest to study whether such ohnologs might have diversified in function from their ancestors as might be indicated by difference in expression patterns. InParanoid [35] was used to generate an orthology mapping between carp and zebrafish. This resulted in 18241 ortholog groups that are presented in Additional file 6. From this orthology mapping we selected 3549 groups for 1 to 2 mapping between zebrafish and carp. This group probably represents a majority of putative ohnologs that are the results of the carp-specific duplication. This group of putative ohnolog gene pairs has been linked to the expression data of 15 different tissues (Additional file 7: Table S3). In Additional file 8: Figure S4 a numerical overview is given of differential expression values at an absolute fold change of two and four for this set of 3549 gene pairs. The results show that in all tissues analyzed over 30 % of the gene pairs show an absolute FC change difference of more than 2. Even at fold chance 4 cut off many pairs are also differentially expressed in a majority of all organs, e.g. four gene pairs are even differentially expressed at FC 4 in all 15 tissues under consideration (Additional file 8: Figure S4). These data suggest that that many putative ohnologs have undergone an evolutionary adaptation either towards being less dominant or alternative functions in various tissues.

### Integrative data viewer

**Fig. 4:**
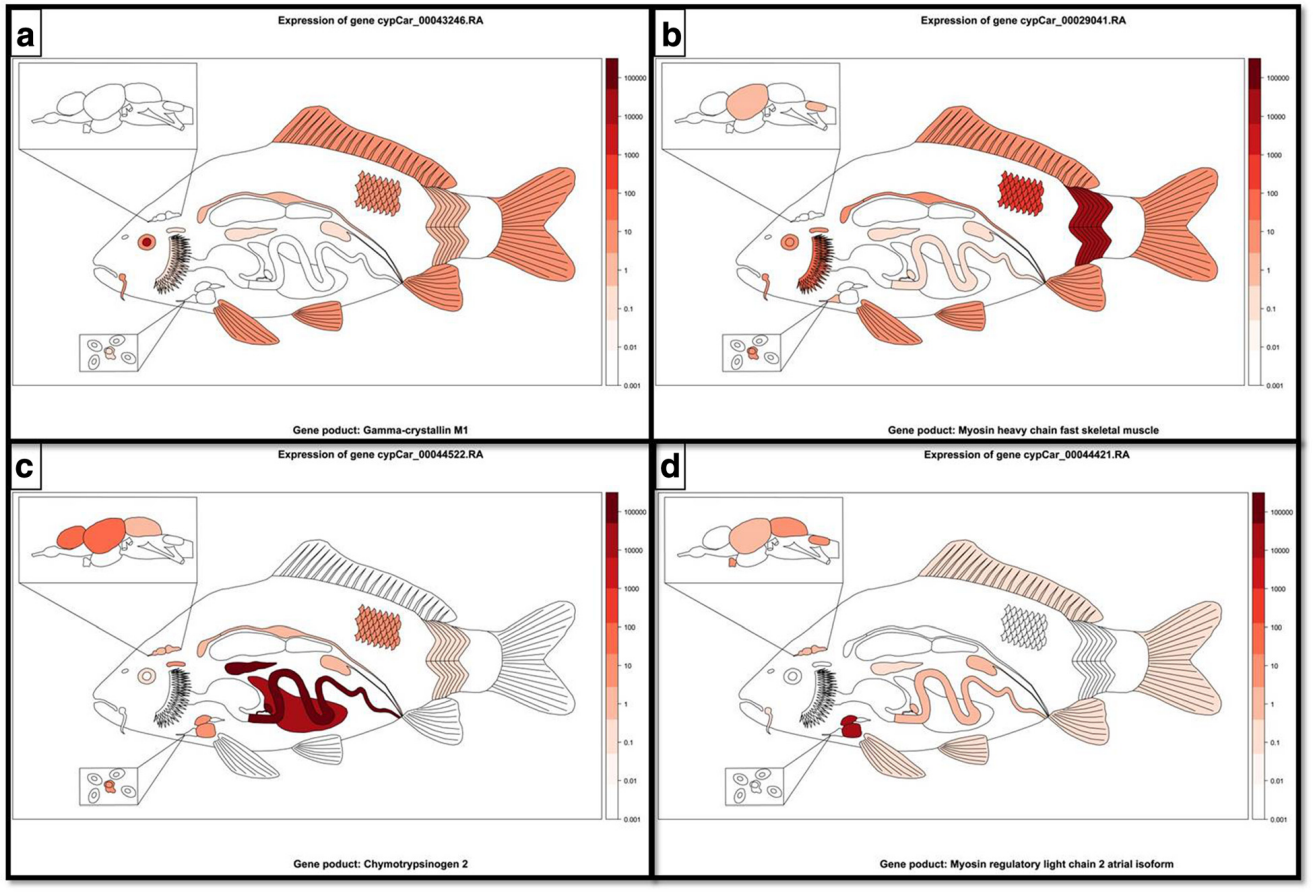
Tissue - specific gene expression visualization in the carp. As an example of this visualization tool, a gene was selected from all four clusters (cluster 6-lens, 11-muscle, 12-heart, and 15-gallbladder) depicted in Fig. 3. For each gene the RPKM-normalized data (log10) was used. **a** depicts the carp gene gamma-crystallin M1 (cypCar_00043246) from cluster 6. Gamma-crystalin M1 is one of the three genes in the carp gamma crystalin gene family. **b** depicts the carp gene myosin heavy chain fast skeletal muscle (cypCar_00029041) from cluster 1. **c** depicts the carp gene chymotrypsinogen 2 (cypCar_00044522) from cluster 15. **d** depicts the carp gene myosin regulatory light chain 2 atrial isoform (cypCar_00044421) from cluster 12

We selected one gene from the top 10 genes from cluster 6, 11, 12 and 15. We used these genes to visualize our transcriptome data in a cartoon representation of the carp body plan using R (Fig. 4). The R script maps the gene and protein expression to individual organs of the carp in a lateral view to obtain a quantitative overview of the organ/organ subpart/tissue when data from an organ pair or from repeated dissections are present. In this manuscript several examples are given, the others can be generated by using the supplementary R script (Additional file 10) and data files (Additional file 9). We are currently working on a web site that will use this script to make it possible to analyze our data in an interactive viewer that will be accessible via the following web address: http://ms-utils.org/comics/.

### Proteome analysis

**Fig. 5:**
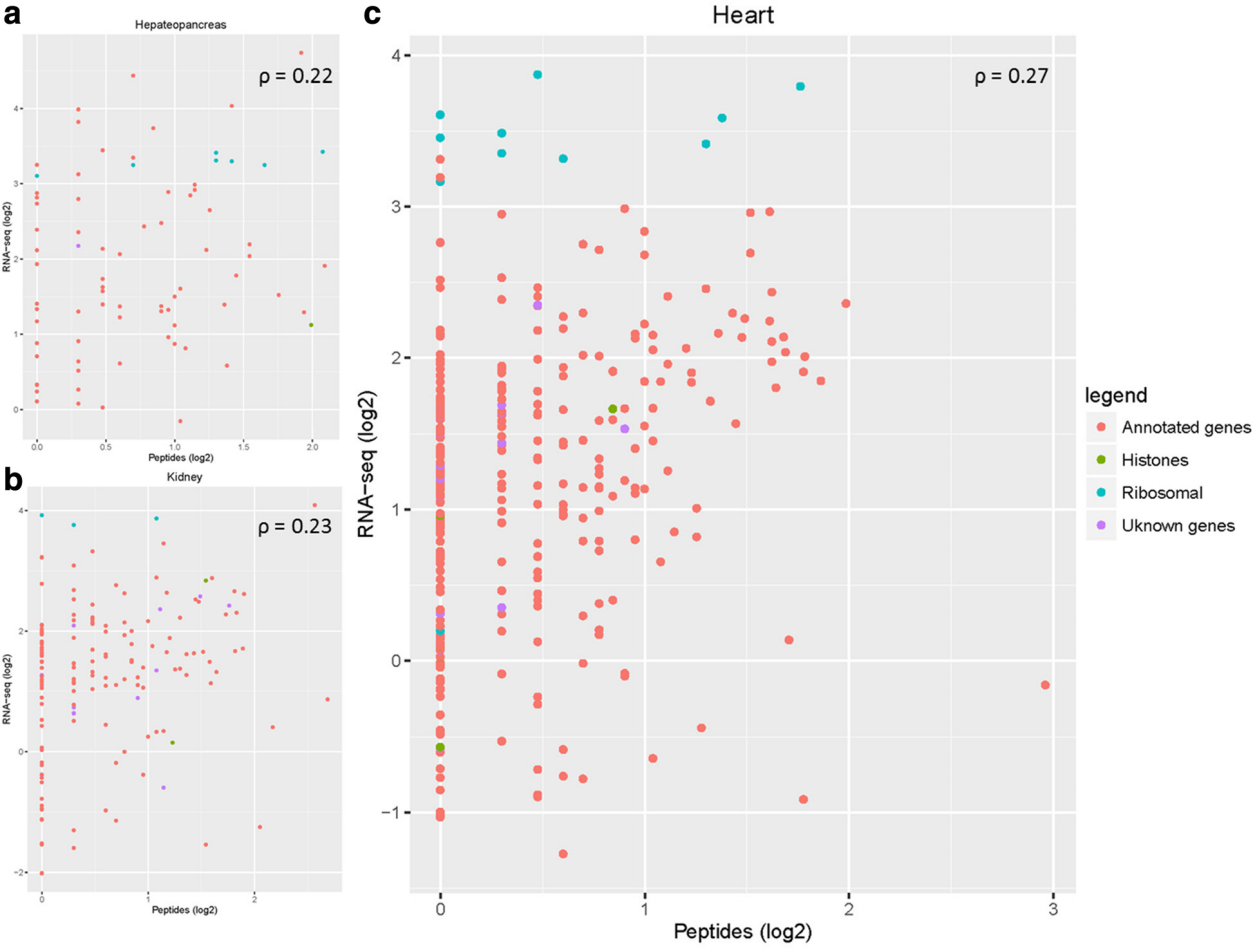
Correlation plots of the RPKM-normalized RNA-Seq and proteins detected data in different carp organs (log2). **a** depicts hepatopancreas tissue. **b** depicts Kidney tissue. **c** depicts heart tissue. The correlation is calculated by using a Spearman correlation test (correlation coefficient -ρ). The genes are colored according four categories: annotated genes (*orange*), histones (*green*), ribosomal (*blue*) and unknown/unannotated genes (*purple*)

For three tissues types (heart, kidney, and hepatopancreas), a proteomics experiment resulted in the identification of 1912 peptides which included 936 uniquely mapped peptides, which were correlated to previously determined RNA levels of these tissues. In total 896 of these peptides were annotated, including 11 histone and 17 ribosomal genes, and a few unknown (Fig. 5). The correlation between the uniquely mapped peptides and RNA-Seq of each tissue individually were calculated with the Spearman correlation coefficient (ρ). This resulted in a moderate correlation between the two datasets for each organ. The correlations are plotted per tissue type on a log2 scale (Fig. 5). The most challenging part was that the majority of proteins were supported by more than one unique peptide. So by selecting only the uniquely mapped peptides for the correlation we lost some power in our analysis, but our results are very similar to those found in zebrafish larvae [36].

These data confirm an earlier report on a comparative parallel transcriptome and proteome analysis of embryonic zebrafish samples [36]. In this study embryonic gene expression was found to correlate with the abundance of proteins with the exception of some classes of proteins such as ribosomal proteins, histones and vitellogenins. Since in several carp organs the expressions of these classes of proteins are variable, the correlation between the transcriptome and proteome data is also variable. However, in general, the measured correlations provide confidence in the analyzed genes and give a good prediction whether they can be used as markers genes at the RNA and / or protein levels in future detailed analyses. More detailed analysis of mRNA-Protein results using approaches such as described by Koussounadis et al. [59] and Cheng et al. [60] would be desirable in future studies of our data sets.

## Conclusions

In the present study, we have compiled an extensive dataset on tissue-specific expression and translation of genes in the European common carp. These data provide a detailed, sensitive and replicable account of gene activity across tissues. We expect these data to be of use for both carp and zebrafish research, as the vast majority of tissue-specific genes are common to both species. Therefore, the carp data will enable the development of gene expression markers specific for selected organs. Our simple expression viewer will support rapid scanning for such markers. As it can be quickly modified to accommodate additional data, the application can be applied to gene comparison with other cyprinid species, which all share a similar body plan. As an increasing number of cyprinid fish genomes have now been sequenced, this opens up promising opportunities for comparative genomic analyses of evolutionary and developmental biology. In summary, therefore, our combined genome, transcriptome and proteome data of one and the same common carp strain will be a valuable resource for future comparative genomics. Furthermore, these data sets and annotations will further enhance the usefulness of common carp for functional genomic studies.

## Additional files

Additional file 1: Table S1. List of the 89 dissected tissues from 19 organs from adult Carps (1 male, 3 female) and their read counts. Total read count was 512.134.436 averaging ~5.754.320 reads per sample. Additionally two 5 dpf embryos were sampled. (XLSX 12 kb) Additional file 2: Figure S1. Description of data: Density plots of the normalized RNA-Seq count data (log10). A: depicts the data normalized using CPM. B: depicts the data normalized using RPKM. C: depicts the rank data normalized using CPM. D: depicts the rank data normalized using RPKM. (PNG 147 kb) Additional file 3: Figure S2. Correlation plot using the Pearson correlation test on the RPKM normalized data (*n* = 91). The different tissues were colored according to the 16 groups. (PDF 198 kb) Additional file 4: Table S2. Description of data: Overview of the division of the tissues (*n* = 87) in the 16 clusters. Clusters are based on the correlation between the samples (Fig. 2). They consist out of biological replicates or closely related tissues. (XLSX 11 kb) Additional file 5: Figure S3. Heatmap on the RPKM-normalized data for all the 16 tissue clusters. All the clusters are visualized, depicting the top 10 highest expressed genes in that respective cluster. The different tissues were colored according to the 16 groups. The clusters were ordered to match the order of the tissue groups depicted in the columns. (PDF 982 kb) Additional file 6: FASTA file containg a set of 18241 ortholog groups that were obtained using InParanoid [35] for orthologs mapping between carp and zebrafish. (FA 1253 kb) Additional file 7: Table S3. Carp putative ohnolog groups linked to expression data in 15 carp tissues. From 18241 carp ortholog groups mapped to zebrafish that are presented in the Additional file 6. we selected 3549 groups for 1 to 2 mapping between zebrafish and carp. This group of putative ohnolog gene pairs have been linked to the expression data of 15 different tissues. A numerical overview of this dataset is presented in Additional file 8: Figure S4. (XLSX 2042 kb) Additional file 8: Figure S4. Numeric overview of differential expression levels of possible paralog pairs. (A) Number of paralog pairs that are differentially expressed with a FC of larger than 2 in 15 separate tissues. (B) Number of possible paralog pairs filtered on the minimum number of tissues in which a differential expression of larger than FC 2 is observed. E.g. in the bottom of the scale 27 possible paralog pairs have a FC of larger than 2 in all 15 tissue types listed in panel A. (C and D) The same analysis as in panels A and B respectively with a FC of 4 as cut-off value. With these criteria there are still 4 possible paralog pairs that have a FC larger than 4 in all 15 tissues. (JPG 130 kb) Additional file 9: Visualisations of carp organ data. Visualisations that can be used with the supplied R-script. (ZIP 2148 kb) Additional file 10: Carp transcriptome expression visualization tool containing the R-script and input files needed to create these visualization. This includes the carp shape file and RPKM normalized(log10) data for all the proteins measured. (ZIP 7041 kb)

## Abbreviations

CLIP: Cre-LoxP Inverse PCR
RNAseq (RNA-seq): RNA deep sequencing
