# Supplementary figures and images for: A full-body transcriptome and proteome resource for the European common carp
Source: BMC Genomics. 2016 Sep 2;17(1):701. doi: 10.1186/s12864-016-3038-y (PMC5009708; doi:10.1186/s12864-016-3038-y)

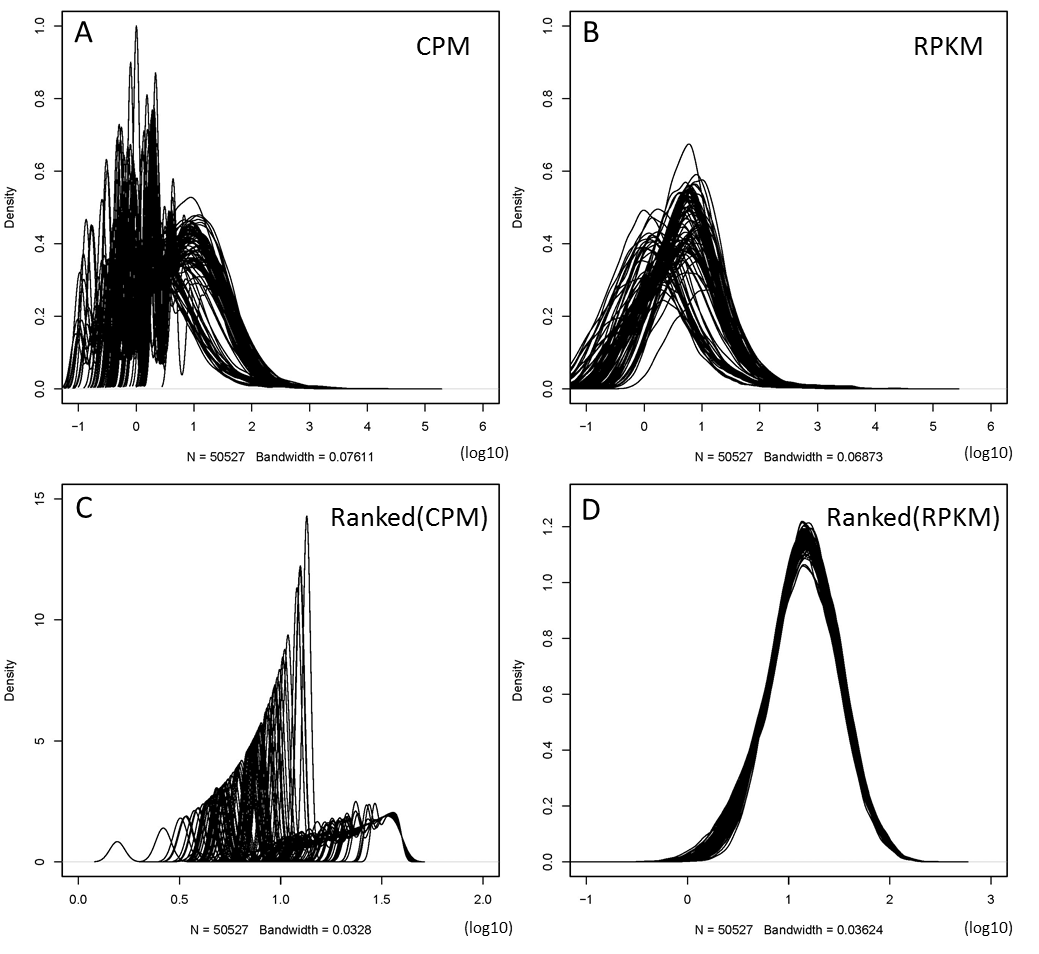

Supplement: Additional file 2: Figure S1. — Description of data: Density plots of the normalized RNA-Seq count data (log10). A: depicts the data normalized using CPM. B: depicts the data normalized using RPKM. C: depicts the rank data normalized using CPM. D: depicts the rank data normalized using RPKM. (PNG 147 kb) [file 12864_2016_3038_MOESM2_ESM.png]

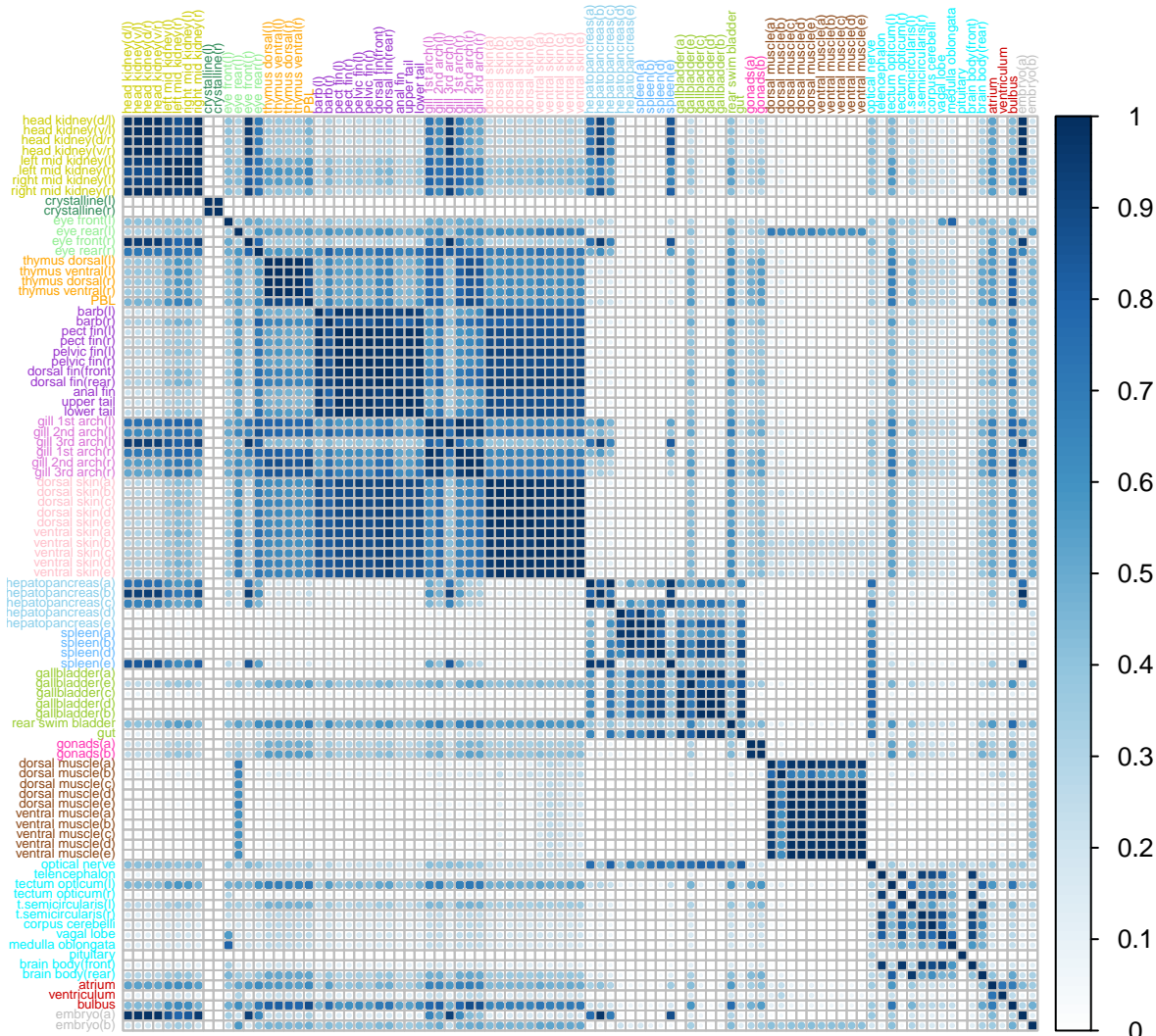

Supplement: Additional file 3: Figure S2. — Correlation plot using the Pearson correlation test on the RPKM normalized data (n = 91). The different tissues were colored according to the 16 groups. (PDF 198 kb) [file 12864_2016_3038_MOESM3_ESM.pdf]

## Heatmap clusters

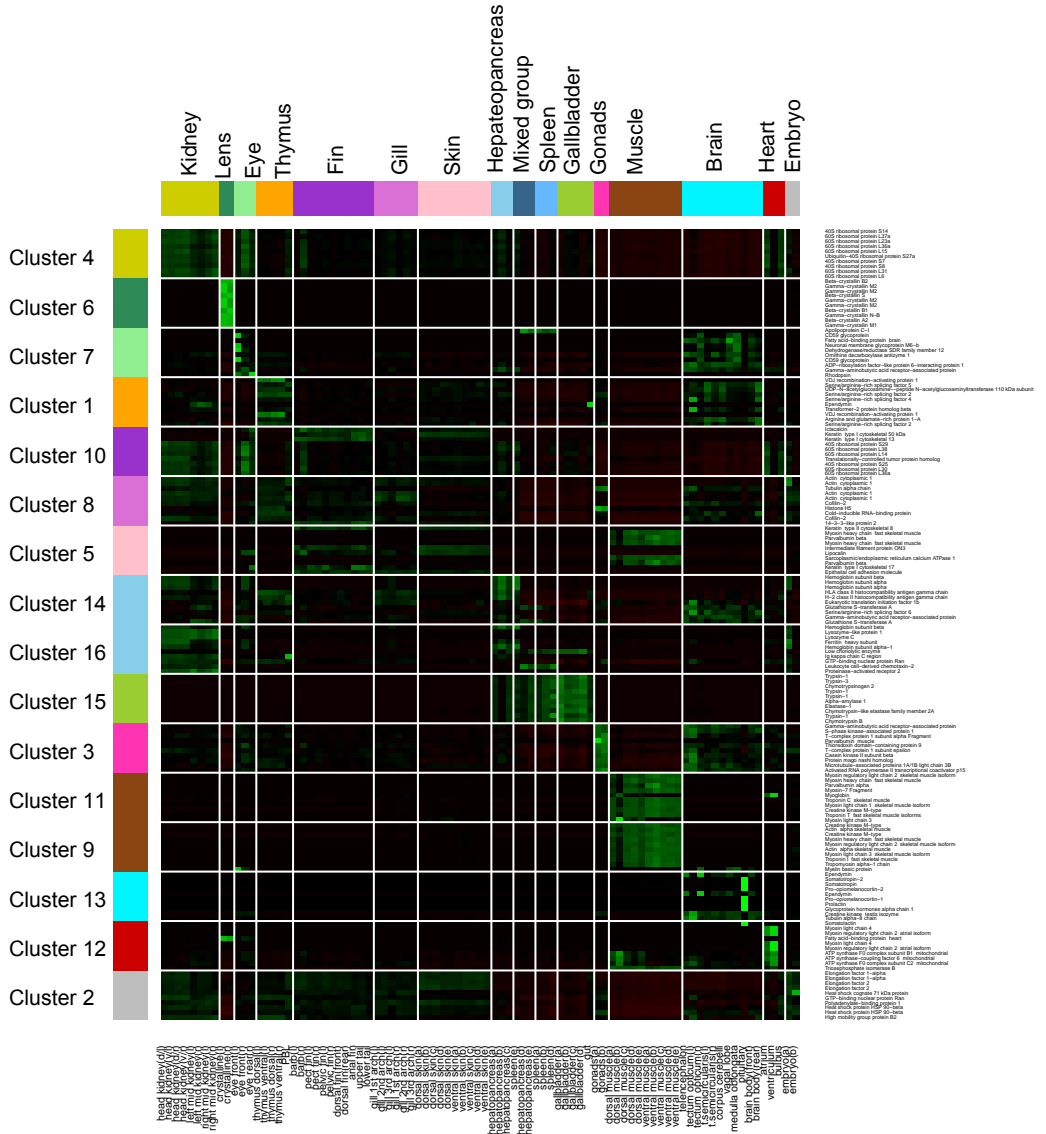

Supplement: Additional file 5: Figure S3. — Heatmap on the RPKM-normalized data for all the 16 tissue clusters. All the clusters are visualized, depicting the top 10 highest expressed genes in that respective cluster. The different tissues were colored according to the 16 groups. The clusters were ordered to match the order of the tissue groups depicted in the columns. (PDF 982 kb) [file 12864_2016_3038_MOESM5_ESM.pdf]

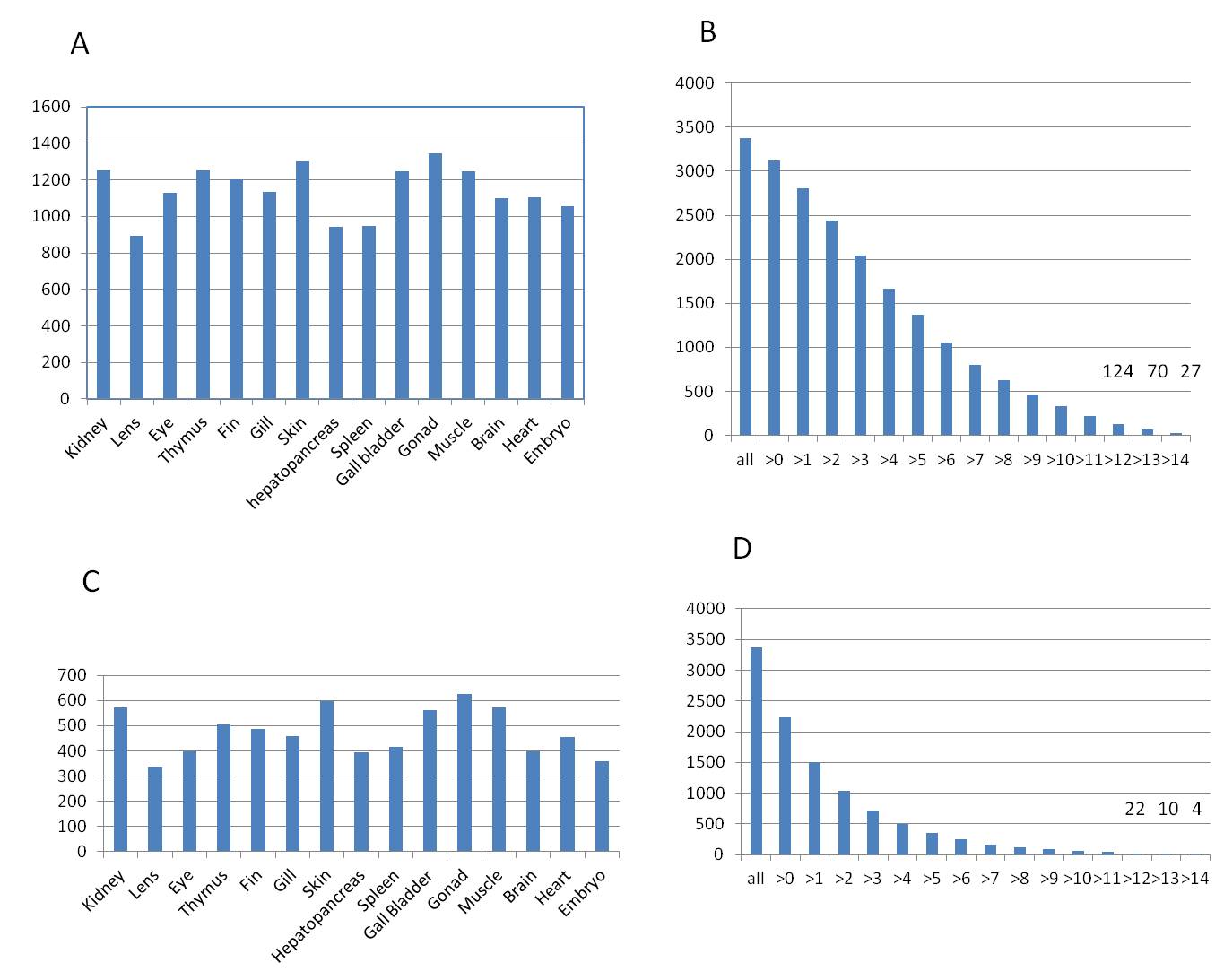

Supplement: Additional file 8: Figure S4. — Numeric overview of differential expression levels of possible paralog pairs. (A) Number of paralog pairs that are differentially expressed with a FC of larger than 2 in 15 separate tissues. (B) Number of possible paralog pairs filtered on the minimum number of tissues in which a differential expression of larger than FC 2 is observed. E.g. in the bottom of the scale 27 possible paralog pairs have a FC of larger than 2 in all 15 tissue types listed in panel A. (C and D) The same analysis as in panels A and B respectively with a FC of 4 as cut-off value. With these criteria there are still 4 possible paralog pairs that have a FC larger than 4 in all 15 tissues. (JPG 130 kb) [file 12864_2016_3038_MOESM8_ESM.jpg]

# Expression of gene: cypCar\_00002601-RA

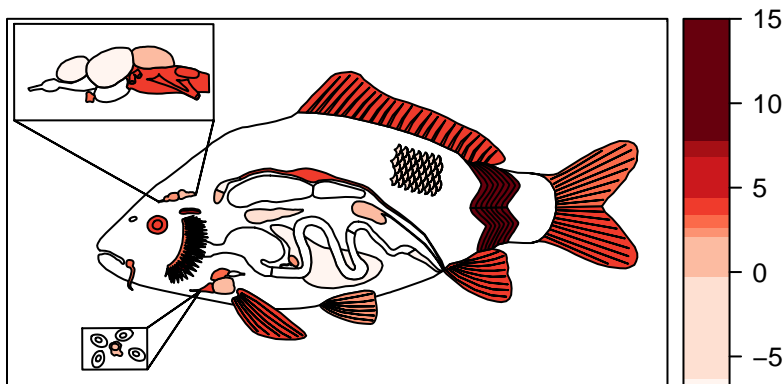

Supplement: Additional file 9: — Visualisations of carp organ data. Visualisations that can be used with the supplied R-script. (ZIP 2148 kb) [file 12864_2016_3038_MOESM9_ESM.zip › carp_final organ viewer/visualizations/cypCar_00002601-RA.pdf]

# Expression of gene: cypCar\_00003591-RA

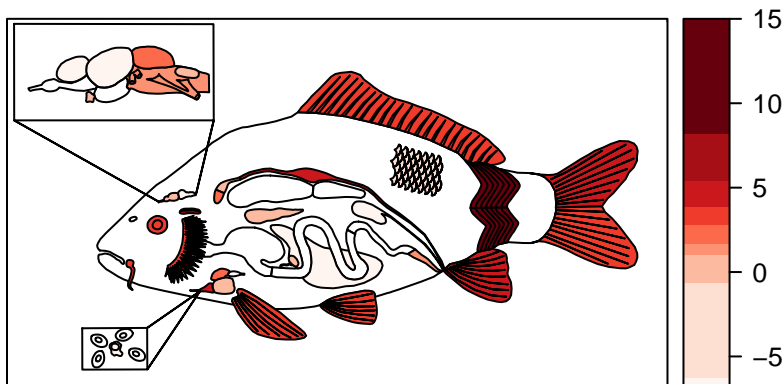

Supplement: Additional file 9: — Visualisations of carp organ data. Visualisations that can be used with the supplied R-script. (ZIP 2148 kb) [file 12864_2016_3038_MOESM9_ESM.zip › carp_final organ viewer/visualizations/cypCar_00003591-RA.pdf]

# Expression of gene: cypCar\_00006488-RA

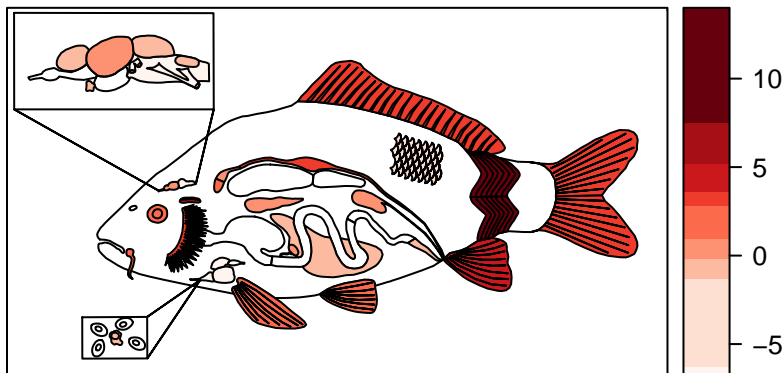

Supplement: Additional file 9: — Visualisations of carp organ data. Visualisations that can be used with the supplied R-script. (ZIP 2148 kb) [file 12864_2016_3038_MOESM9_ESM.zip › carp_final organ viewer/visualizations/cypCar_00006488-RA.pdf]

# Expression of gene: cypCar\_00006626-RA

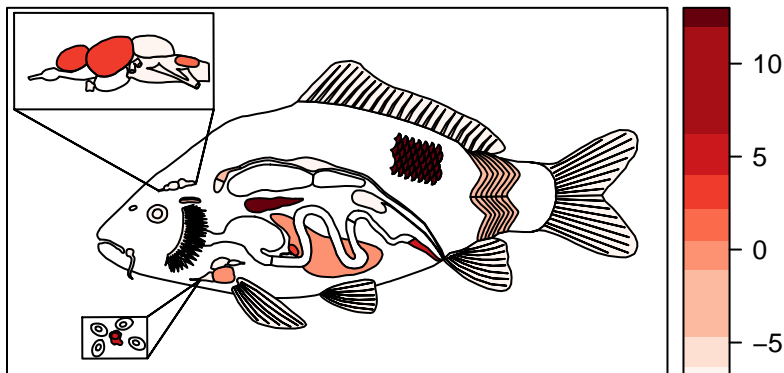

Supplement: Additional file 9: — Visualisations of carp organ data. Visualisations that can be used with the supplied R-script. (ZIP 2148 kb) [file 12864_2016_3038_MOESM9_ESM.zip › carp_final organ viewer/visualizations/cypCar_00006626-RA.pdf]

# Expression of gene: cypCar\_00008824-RA

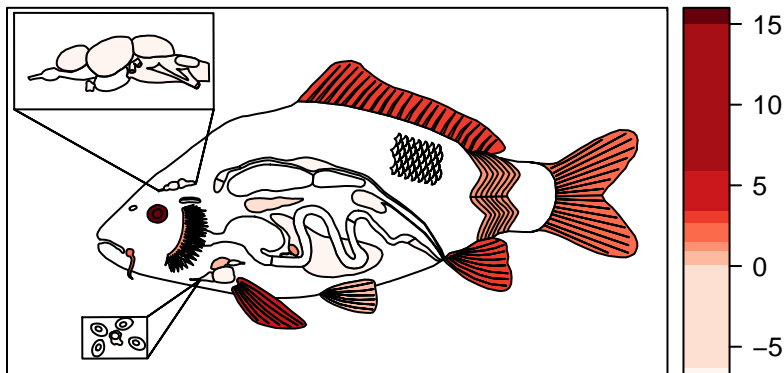

Supplement: Additional file 9: — Visualisations of carp organ data. Visualisations that can be used with the supplied R-script. (ZIP 2148 kb) [file 12864_2016_3038_MOESM9_ESM.zip › carp_final organ viewer/visualizations/cypCar_00008824-RA.pdf]

# Expression of gene: cypCar\_00009709-RA

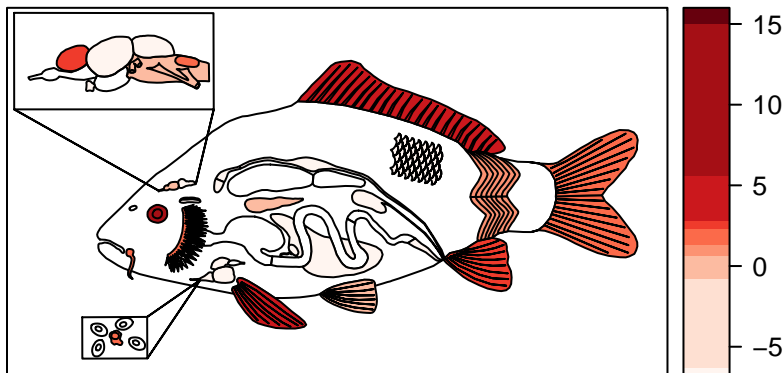

Supplement: Additional file 9: — Visualisations of carp organ data. Visualisations that can be used with the supplied R-script. (ZIP 2148 kb) [file 12864_2016_3038_MOESM9_ESM.zip › carp_final organ viewer/visualizations/cypCar_00009709-RA.pdf]

# Expression of gene: cypCar\_00010835-RA

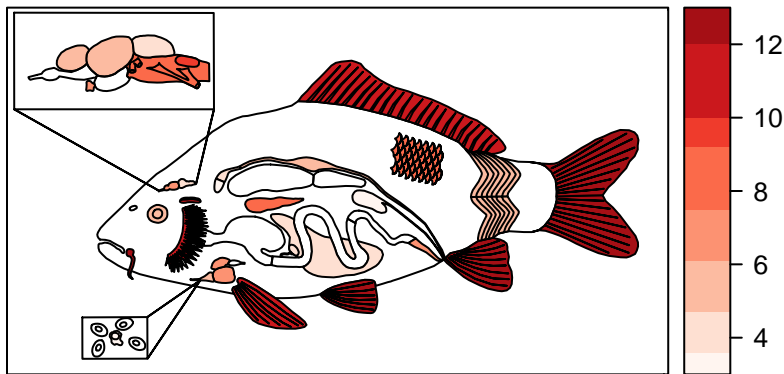

Supplement: Additional file 9: — Visualisations of carp organ data. Visualisations that can be used with the supplied R-script. (ZIP 2148 kb) [file 12864_2016_3038_MOESM9_ESM.zip › carp_final organ viewer/visualizations/cypCar_00010835-RA.pdf]

# Expression of gene: cypCar\_00011327-RA

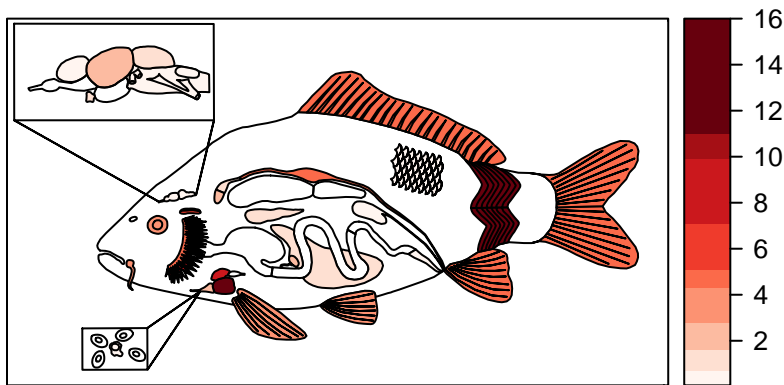

Supplement: Additional file 9: — Visualisations of carp organ data. Visualisations that can be used with the supplied R-script. (ZIP 2148 kb) [file 12864_2016_3038_MOESM9_ESM.zip › carp_final organ viewer/visualizations/cypCar_00011327-RA.pdf]

# Expression of gene: cypCar\_00012466-RA

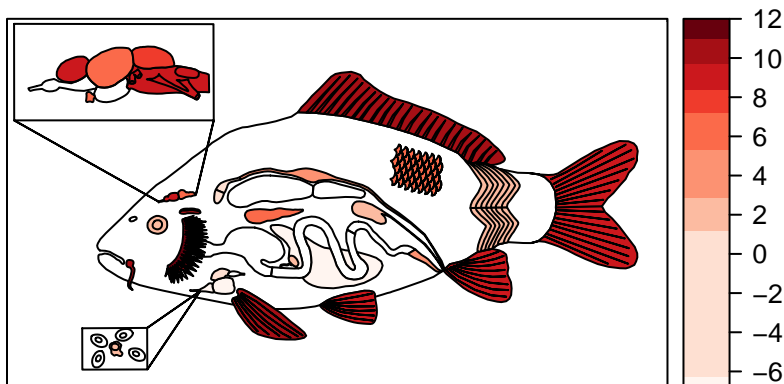

Supplement: Additional file 9: — Visualisations of carp organ data. Visualisations that can be used with the supplied R-script. (ZIP 2148 kb) [file 12864_2016_3038_MOESM9_ESM.zip › carp_final organ viewer/visualizations/cypCar_00012466-RA.pdf]

# Expression of gene: cypCar\_00013291-RA

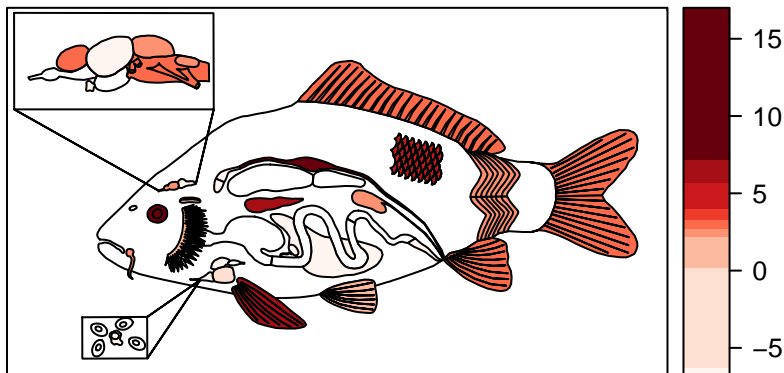

Supplement: Additional file 9: — Visualisations of carp organ data. Visualisations that can be used with the supplied R-script. (ZIP 2148 kb) [file 12864_2016_3038_MOESM9_ESM.zip › carp_final organ viewer/visualizations/cypCar_00013291-RA.pdf]

# Expression of gene: cypCar\_00014609-RA

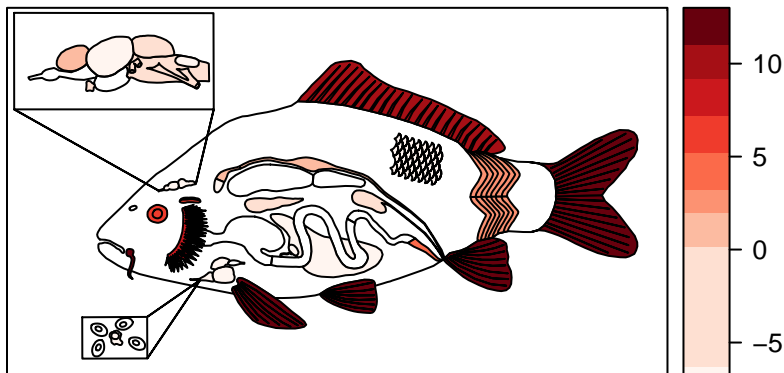

Supplement: Additional file 9: — Visualisations of carp organ data. Visualisations that can be used with the supplied R-script. (ZIP 2148 kb) [file 12864_2016_3038_MOESM9_ESM.zip › carp_final organ viewer/visualizations/cypCar_00014609-RA.pdf]

# Expression of gene: cypCar\_00015772-RA

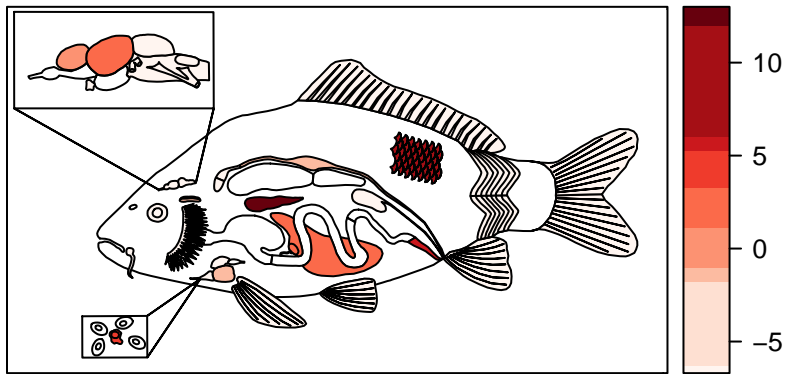

Supplement: Additional file 9: — Visualisations of carp organ data. Visualisations that can be used with the supplied R-script. (ZIP 2148 kb) [file 12864_2016_3038_MOESM9_ESM.zip › carp_final organ viewer/visualizations/cypCar_00015772-RA.pdf]

# Expression of gene: cypCar\_00019640-RA

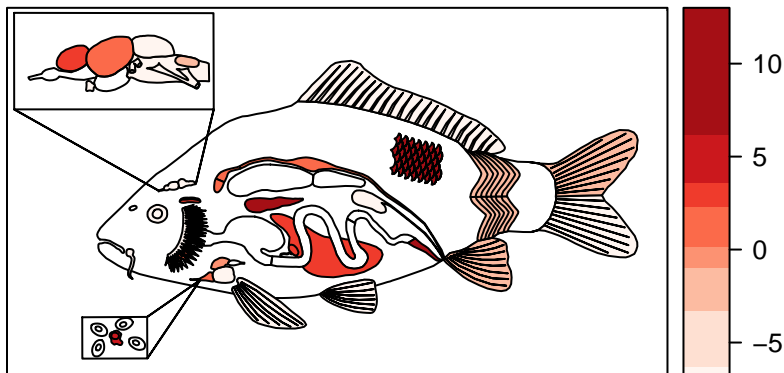

Supplement: Additional file 9: — Visualisations of carp organ data. Visualisations that can be used with the supplied R-script. (ZIP 2148 kb) [file 12864_2016_3038_MOESM9_ESM.zip › carp_final organ viewer/visualizations/cypCar_00019640-RA.pdf]

# Expression of gene: cypCar\_00021815-RA

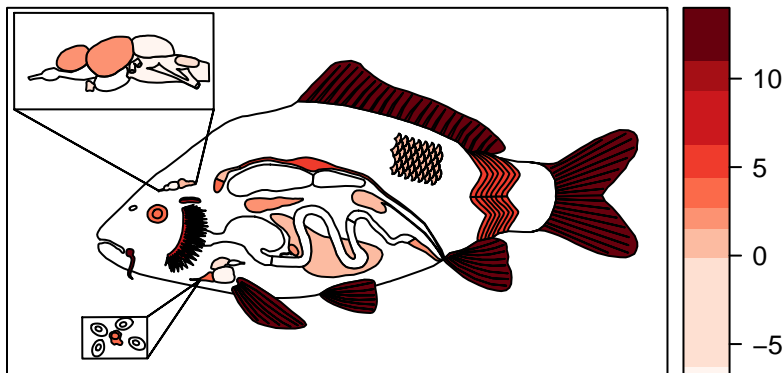

Supplement: Additional file 9: — Visualisations of carp organ data. Visualisations that can be used with the supplied R-script. (ZIP 2148 kb) [file 12864_2016_3038_MOESM9_ESM.zip › carp_final organ viewer/visualizations/cypCar_00021815-RA.pdf]

# Expression of gene: cypCar\_00022052-RA

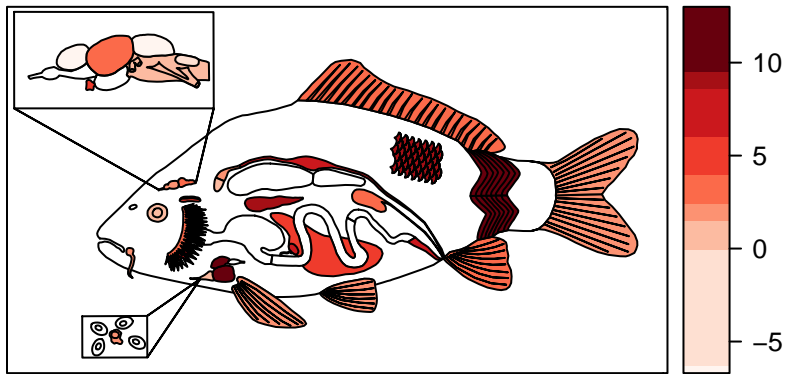

Supplement: Additional file 9: — Visualisations of carp organ data. Visualisations that can be used with the supplied R-script. (ZIP 2148 kb) [file 12864_2016_3038_MOESM9_ESM.zip › carp_final organ viewer/visualizations/cypCar_00022052-RA.pdf]

# Expression of gene: cypCar\_00022420-RA

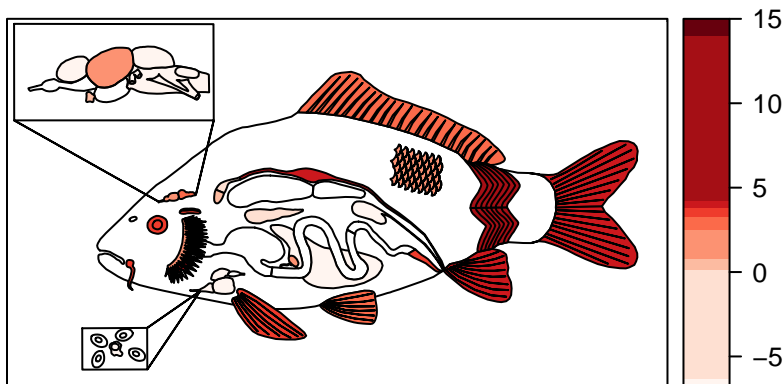

Supplement: Additional file 9: — Visualisations of carp organ data. Visualisations that can be used with the supplied R-script. (ZIP 2148 kb) [file 12864_2016_3038_MOESM9_ESM.zip › carp_final organ viewer/visualizations/cypCar_00022420-RA.pdf]

# Expression of gene: cypCar\_00025600-RA

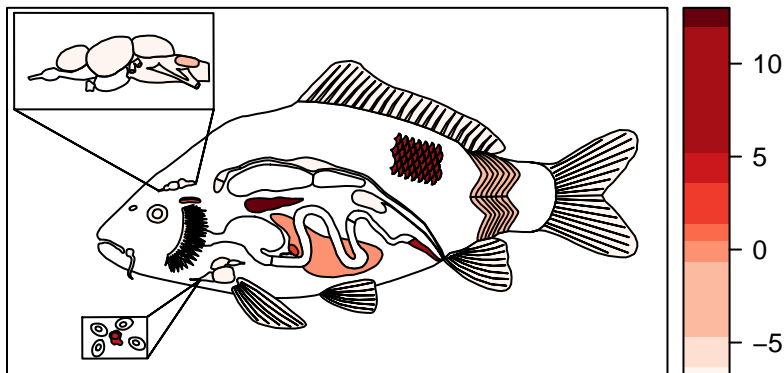

Supplement: Additional file 9: — Visualisations of carp organ data. Visualisations that can be used with the supplied R-script. (ZIP 2148 kb) [file 12864_2016_3038_MOESM9_ESM.zip › carp_final organ viewer/visualizations/cypCar_00025600-RA.pdf]

# Expression of gene: cypCar\_00027580-RA

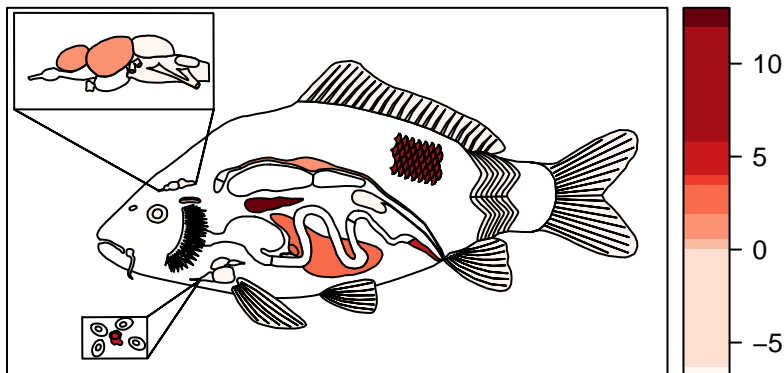

Supplement: Additional file 9: — Visualisations of carp organ data. Visualisations that can be used with the supplied R-script. (ZIP 2148 kb) [file 12864_2016_3038_MOESM9_ESM.zip › carp_final organ viewer/visualizations/cypCar_00027580-RA.pdf]

# Expression of gene: cypCar\_00029040-RA

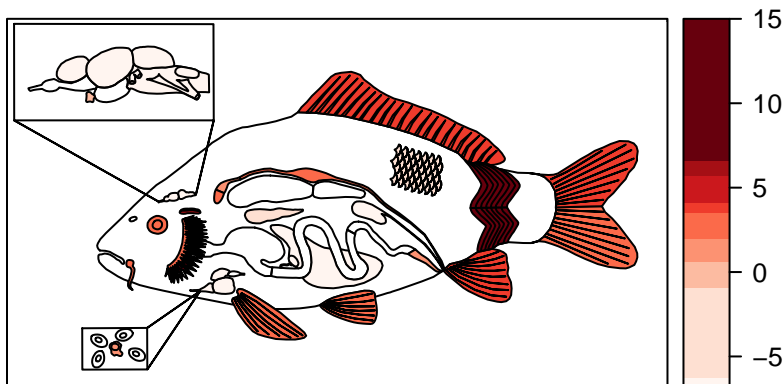

Supplement: Additional file 9: — Visualisations of carp organ data. Visualisations that can be used with the supplied R-script. (ZIP 2148 kb) [file 12864_2016_3038_MOESM9_ESM.zip › carp_final organ viewer/visualizations/cypCar_00029040-RA.pdf]

# Expression of gene: cypCar\_00029041-RA

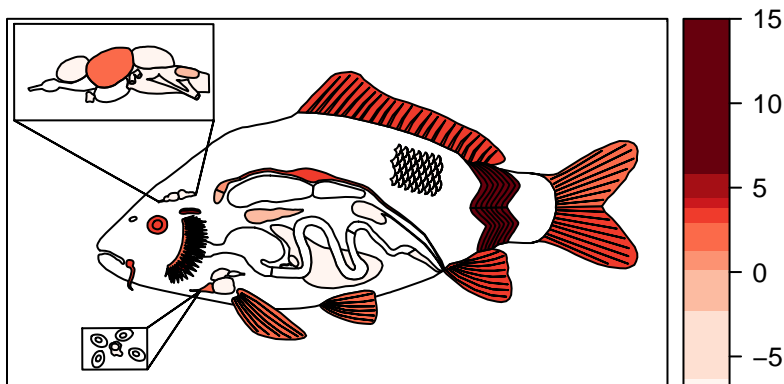

Supplement: Additional file 9: — Visualisations of carp organ data. Visualisations that can be used with the supplied R-script. (ZIP 2148 kb) [file 12864_2016_3038_MOESM9_ESM.zip › carp_final organ viewer/visualizations/cypCar_00029041-RA.pdf]

# Expression of gene: cypCar\_00029514-RA

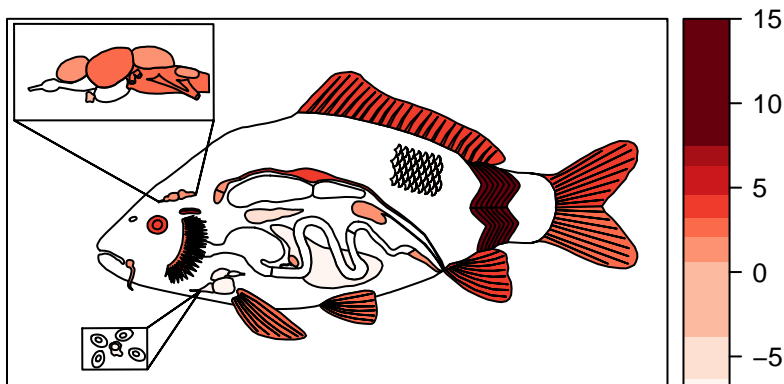

Supplement: Additional file 9: — Visualisations of carp organ data. Visualisations that can be used with the supplied R-script. (ZIP 2148 kb) [file 12864_2016_3038_MOESM9_ESM.zip › carp_final organ viewer/visualizations/cypCar_00029514-RA.pdf]

# Expression of gene: cypCar\_00032715-RA

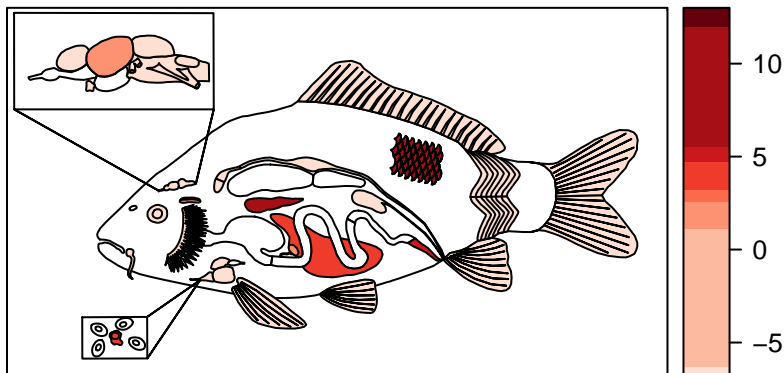

Supplement: Additional file 9: — Visualisations of carp organ data. Visualisations that can be used with the supplied R-script. (ZIP 2148 kb) [file 12864_2016_3038_MOESM9_ESM.zip › carp_final organ viewer/visualizations/cypCar_00032715-RA.pdf]

# Expression of gene: cypCar\_00033086-RA

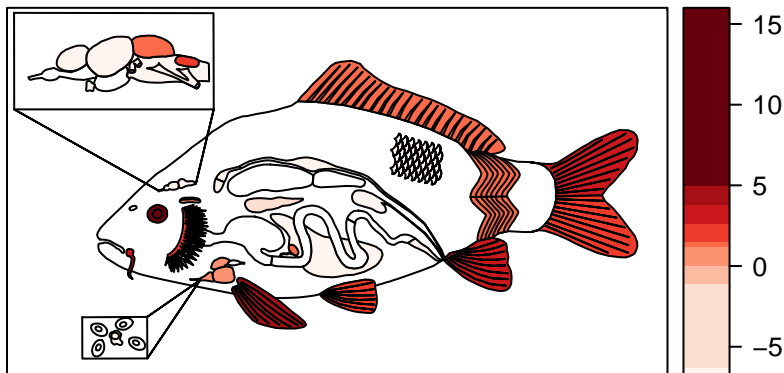

Supplement: Additional file 9: — Visualisations of carp organ data. Visualisations that can be used with the supplied R-script. (ZIP 2148 kb) [file 12864_2016_3038_MOESM9_ESM.zip › carp_final organ viewer/visualizations/cypCar_00033086-RA.pdf]

# Expression of gene: cypCar\_00034481-RA

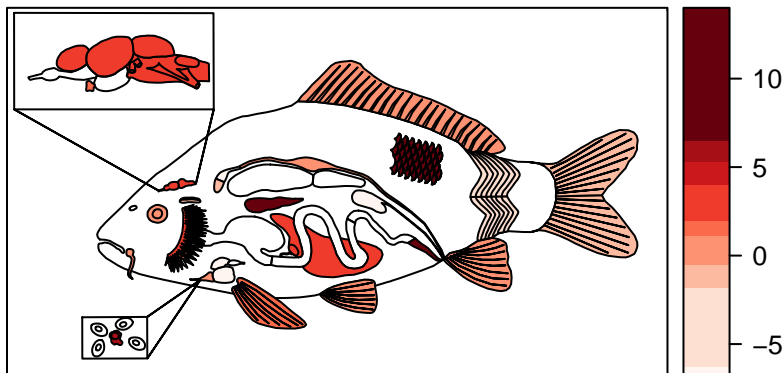

Supplement: Additional file 9: — Visualisations of carp organ data. Visualisations that can be used with the supplied R-script. (ZIP 2148 kb) [file 12864_2016_3038_MOESM9_ESM.zip › carp_final organ viewer/visualizations/cypCar_00034481-RA.pdf]

# Expression of gene: cypCar\_00038482-RA

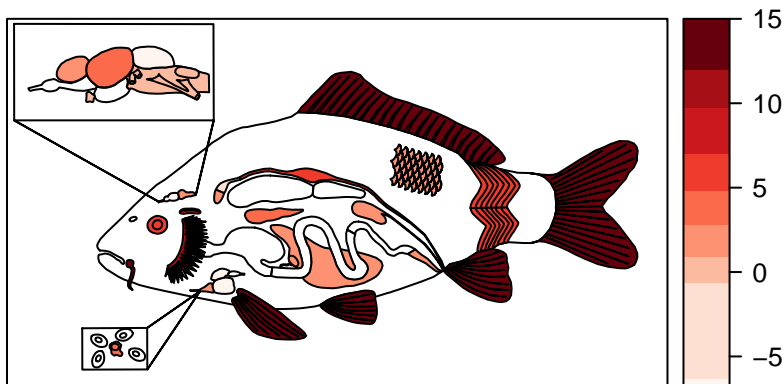

Supplement: Additional file 9: — Visualisations of carp organ data. Visualisations that can be used with the supplied R-script. (ZIP 2148 kb) [file 12864_2016_3038_MOESM9_ESM.zip › carp_final organ viewer/visualizations/cypCar_00038482-RA.pdf]

# Expression of gene: cypCar\_00039271-RA

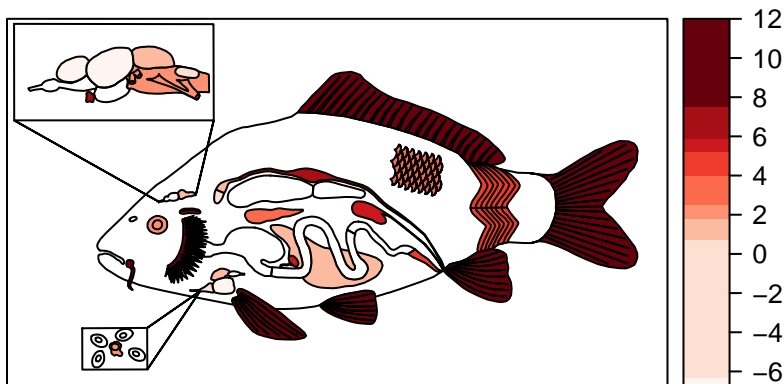

Supplement: Additional file 9: — Visualisations of carp organ data. Visualisations that can be used with the supplied R-script. (ZIP 2148 kb) [file 12864_2016_3038_MOESM9_ESM.zip › carp_final organ viewer/visualizations/cypCar_00039271-RA.pdf]

# Expression of gene: cypCar\_00040488-RA

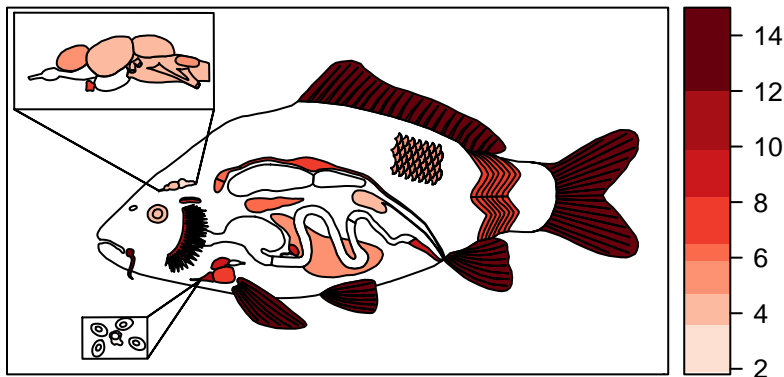

Supplement: Additional file 9: — Visualisations of carp organ data. Visualisations that can be used with the supplied R-script. (ZIP 2148 kb) [file 12864_2016_3038_MOESM9_ESM.zip › carp_final organ viewer/visualizations/cypCar_00040488-RA.pdf]

# Expression of gene: cypCar\_00040548-RA

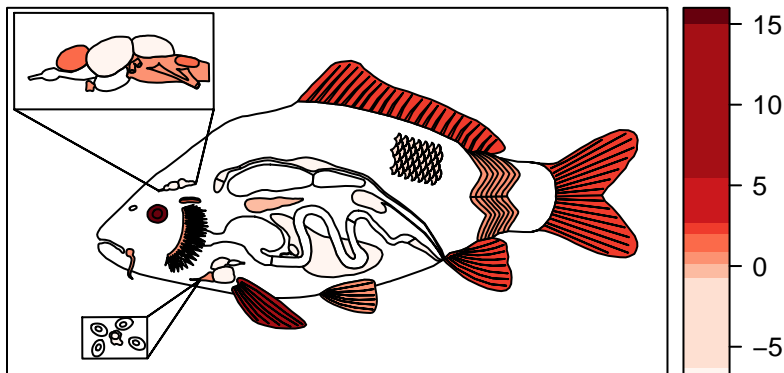

Supplement: Additional file 9: — Visualisations of carp organ data. Visualisations that can be used with the supplied R-script. (ZIP 2148 kb) [file 12864_2016_3038_MOESM9_ESM.zip › carp_final organ viewer/visualizations/cypCar_00040548-RA.pdf]

# Expression of gene: cypCar\_00043246-RA

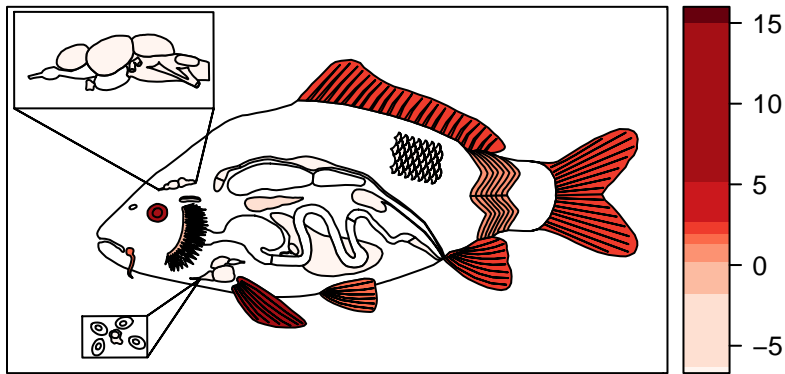

Supplement: Additional file 9: — Visualisations of carp organ data. Visualisations that can be used with the supplied R-script. (ZIP 2148 kb) [file 12864_2016_3038_MOESM9_ESM.zip › carp_final organ viewer/visualizations/cypCar_00043246-RA.pdf]

# Expression of gene: cypCar\_00046269-RA

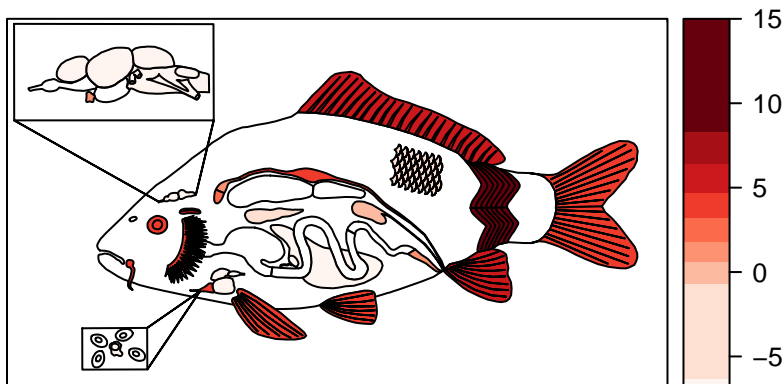

Supplement: Additional file 9: — Visualisations of carp organ data. Visualisations that can be used with the supplied R-script. (ZIP 2148 kb) [file 12864_2016_3038_MOESM9_ESM.zip › carp_final organ viewer/visualizations/cypCar_00046269-RA.pdf]

# Expression of gene: cypCar\_00047519-RA

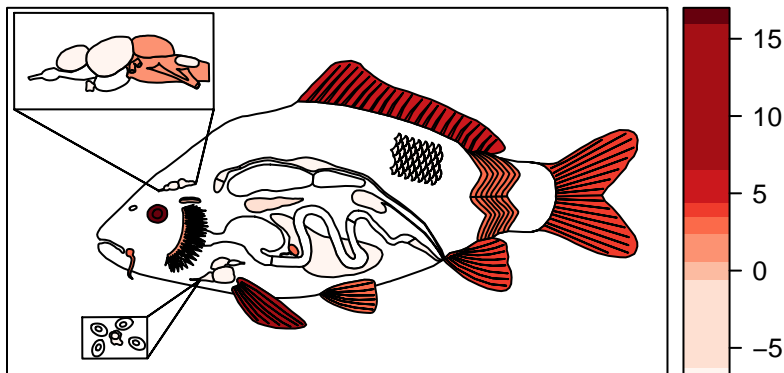

Supplement: Additional file 9: — Visualisations of carp organ data. Visualisations that can be used with the supplied R-script. (ZIP 2148 kb) [file 12864_2016_3038_MOESM9_ESM.zip › carp_final organ viewer/visualizations/cypCar_00047519-RA.pdf]

# Expression of gene: cypCar\_00048311-RA

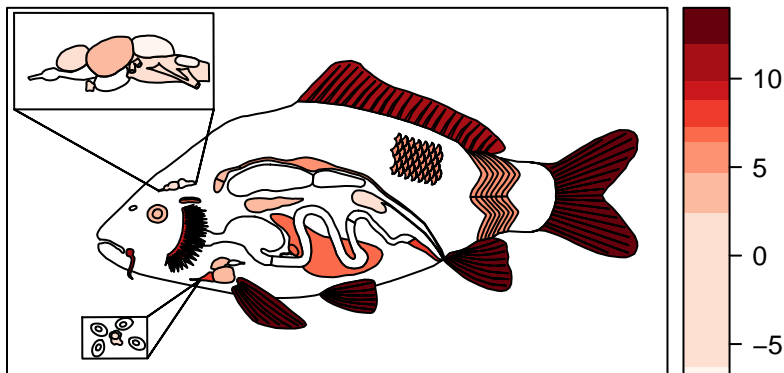

Supplement: Additional file 9: — Visualisations of carp organ data. Visualisations that can be used with the supplied R-script. (ZIP 2148 kb) [file 12864_2016_3038_MOESM9_ESM.zip › carp_final organ viewer/visualizations/cypCar_00048311-RA.pdf]

# Expression of gene: cypCar\_00048739-RA

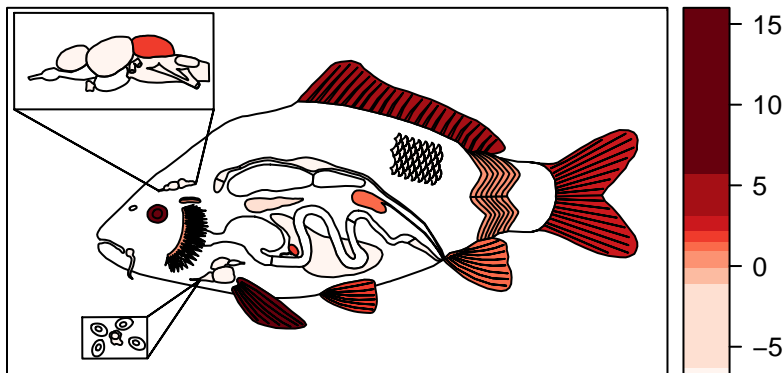

Supplement: Additional file 9: — Visualisations of carp organ data. Visualisations that can be used with the supplied R-script. (ZIP 2148 kb) [file 12864_2016_3038_MOESM9_ESM.zip › carp_final organ viewer/visualizations/cypCar_00048739-RA.pdf]

# Expression of gene: cypCar\_00048865-RA

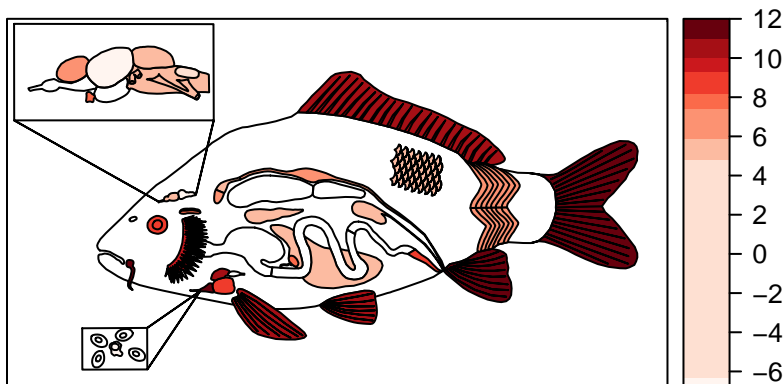

Supplement: Additional file 9: — Visualisations of carp organ data. Visualisations that can be used with the supplied R-script. (ZIP 2148 kb) [file 12864_2016_3038_MOESM9_ESM.zip › carp_final organ viewer/visualizations/cypCar_00048865-RA.pdf]

# Expression of gene: cypCar\_00049166-RA

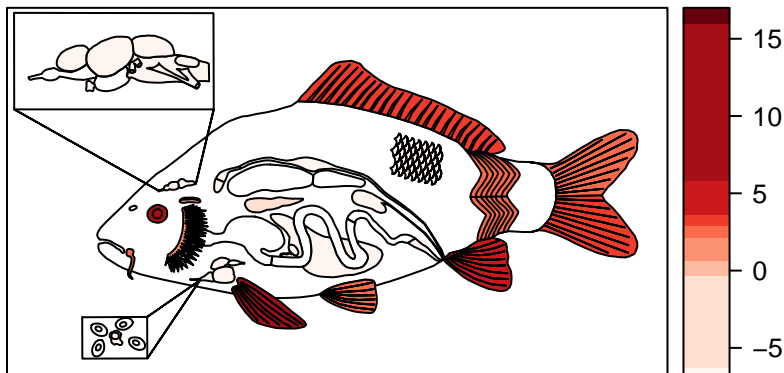

Supplement: Additional file 9: — Visualisations of carp organ data. Visualisations that can be used with the supplied R-script. (ZIP 2148 kb) [file 12864_2016_3038_MOESM9_ESM.zip › carp_final organ viewer/visualizations/cypCar_00049166-RA.pdf]

# Expression of gene: cypCar\_00049316-RA

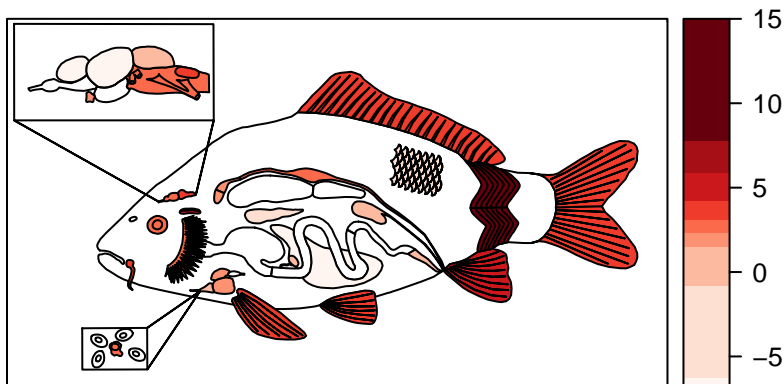

Supplement: Additional file 9: — Visualisations of carp organ data. Visualisations that can be used with the supplied R-script. (ZIP 2148 kb) [file 12864_2016_3038_MOESM9_ESM.zip › carp_final organ viewer/visualizations/cypCar_00049316-RA.pdf]

# Expression of gene: cypCar\_00049417-RA

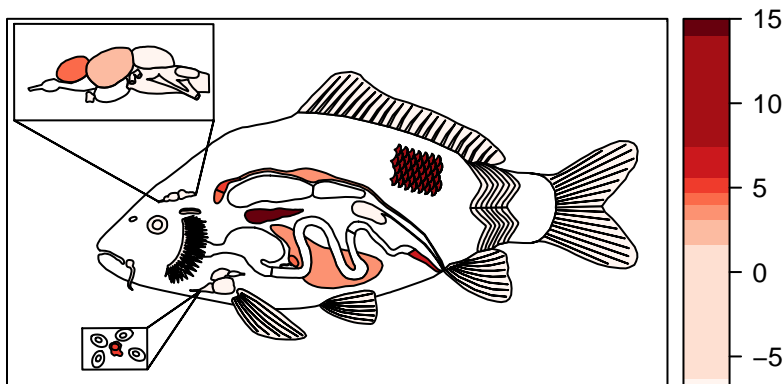

Supplement: Additional file 9: — Visualisations of carp organ data. Visualisations that can be used with the supplied R-script. (ZIP 2148 kb) [file 12864_2016_3038_MOESM9_ESM.zip › carp_final organ viewer/visualizations/cypCar_00049417-RA.pdf]

# Expression of gene: cypCar\_00049712-RA

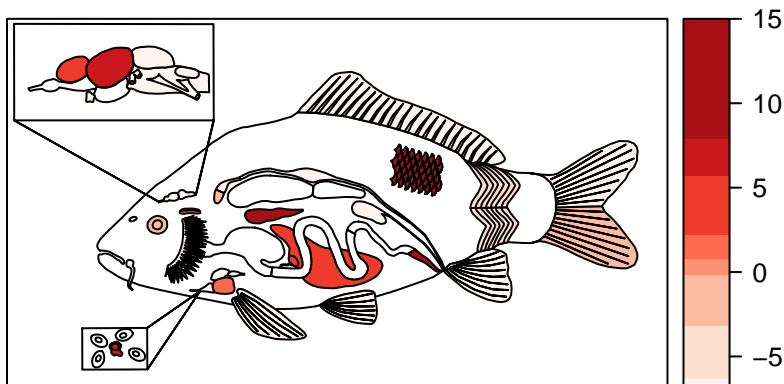

Supplement: Additional file 9: — Visualisations of carp organ data. Visualisations that can be used with the supplied R-script. (ZIP 2148 kb) [file 12864_2016_3038_MOESM9_ESM.zip › carp_final organ viewer/visualizations/cypCar_00049712-RA.pdf]

# Expression of gene: cypCar\_00049824-RA

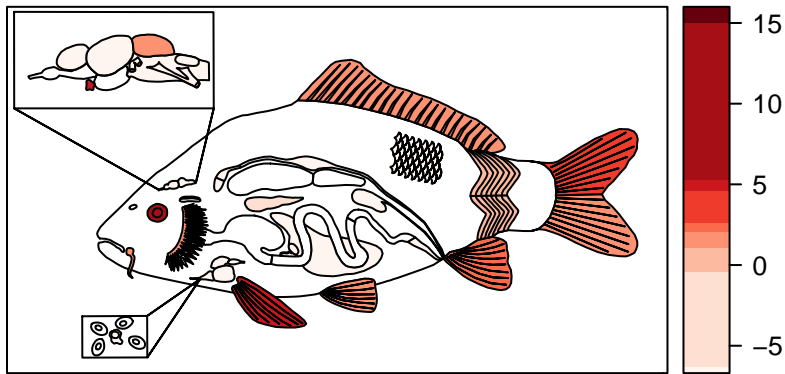

Supplement: Additional file 9: — Visualisations of carp organ data. Visualisations that can be used with the supplied R-script. (ZIP 2148 kb) [file 12864_2016_3038_MOESM9_ESM.zip › carp_final organ viewer/visualizations/cypCar_00049824-RA.pdf]

# Expression of gene: cypCar\_00050443-RA

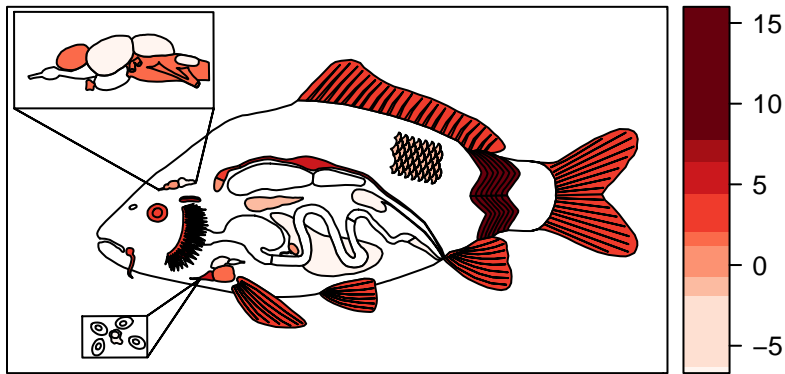

Supplement: Additional file 9: — Visualisations of carp organ data. Visualisations that can be used with the supplied R-script. (ZIP 2148 kb) [file 12864_2016_3038_MOESM9_ESM.zip › carp_final organ viewer/visualizations/cypCar_00050443-RA.pdf]
